# Supplementary material for: Investigating point-of-care diagnostics for sexually transmitted infections and antimicrobial resistance in antenatal care in Zimbabwe (IPSAZ): protocol for a mixed-methods study
Source: BMJ Open. 2023 Apr 20;13(4):e070889. doi: 10.1136/bmjopen-2022-070889 (PMC10124298; doi:10.1136/bmjopen-2022-070889)
Supplement: Supplementary data [file bmjopen-2022-070889supp001.pdf]

BMJ Publishing Group Limited (BMJ) disclaims all liability and responsibility arising from any reliance placed on this supplemental material which has been supplied by the author(s)

MRCZ/A/2899

Investigating point-of-care diagnostics for sexually transmitted infections and antimicrobial resistance in primary care in Zimbabwe (IPSAZ)

STI TESTING

Principal Investigator: Dr Kevin Martin [MBBS, MRCP, MSc, DTM&H, DFSRH]
Phone number(s): +263 (0)774 410908

What you should know about this research study:

- We give you this consent form so that you may read about the purpose, risks, and benefits of this research study.
- Routine care is based upon the best known treatment and is provided with the main goal of helping the individual patient. The main goal of research studies is to gain knowledge that may help future patients.
- We cannot promise that this research will benefit you.
- You have the right to refuse to take part, or agree to take part now and change your mind later.
- Whatever you decide, it will not affect your regular care.
- Please review this consent form carefully. Ask any questions before you make a decision.
- Your participation is voluntary.

PURPOSE

We are conducting a research study to find out if it is feasible to offer testing for multiple sexually transmitted infections (STIs) and mental health screening to pregnant women when they attend clinic for routine care. We also want to know the proportion of pregnant women who have STIs and are at risk of depression.. You have been invited to take part because you are pregnant and attending clinic for antenatal care. We are hoping to recruit about 1,000 pregnant women in Harare into this study.

MEDICAL RESEARCH COUNCIL OF ZIMBABWE
10 DEC 2022
APPROVED
20 CAMBRIDGE ROAD, AVONDALE, HARARE

PROCEDURES AND DURATION

If you decide to participate, we will perform tests for the following STIs; chlamydia; gonorrhoea, trichomoniasis; syphilis; hepatitis B; and HIV. These are all infections that can be transmitted by sex. Syphilis and HIV testing is part of routine pregnancy care. Chlamydia, gonorrhoea, trichomoniasis, and syphilis, can all be cured with antibiotic treatment. If left untreated, these STIs can cause harm to both your baby and yourself. Hepatitis B is a virus that infects the liver, and can lead to liver damage. There is no cure, but specialist treatment may be available. Hepatitis B can be passed on to a baby at birth, but the risk of this can be reduced by giving a hepatitis B vaccine to the baby shortly after birth. HIV is a virus which weakens your immune system and your ability to fight everyday infections and disease. If left untreated, it can lead to AIDS, which is the name used to describe a number of potentially life-threatening infections and illnesses that happen when your immune system has been severely damaged by HIV. HIV can also be passed on to the baby during pregnancy. There's currently no cure for HIV, but there are very effective drug treatments that enable most of those on treatment to live a long and healthy life, to reduce the risk of passing the infection to your baby, and to prevent infection of sexual partners.

To perform the tests, we will require two vaginal swabs, which may be taken by yourself or by a member of the study team. We will also take a small fingerprick blood sample. The results for the trichomoniasis, syphilis, hepatitis B, and HIV tests, should all be ready within 30 minutes. During this time, you will undergo a questionnaire. We will ask questions about yourself, your pregnancy, STIs, your risk of STIs and screen for depression. You may stop at any point if you get tired or the questions make you uncomfortable.

The results for the chlamydia and gonorrhoea tests will take 90 minutes. We will ask you to wait or return later today to receive your chlamydia and gonorrhoea results. If you are unable to return to collect your results, and your results are positive, you will be contacted by telephone and asked to return.

If your results for chlamydia, gonorrhoea, trichomoniasis, or syphilis, are positive, we will offer you treatment free of charge. We will also advise that any sexual partners you have also receive treatment. This is important to both protect your partner, and also to prevent re-infection of you. We will provide a slip of paper you can give to your partner, and discuss any concerns you may have. We will not inform your partner of your results without your permission.

If you are newly diagnosed with HIV, we will follow routine practices at the clinic for referral to specialist services. If you screen positive for depression, will refer you to counselling services.

If you are newly diagnosed with hepatitis B, we will refer you to see a liver specialist. To help the specialist decide on the best management, we will also take a blood sample from the vein to check the levels of hepatitis B, and for liver damage. We will also try to arrange for a hepatitis B vaccine to be administered to your new-born baby, to prevent your baby from getting hepatitis B.

If your results for gonorrhoea are positive, a member of the study team will ask to take a further swab from your cervix, which is inside your vagina. These samples will be taken to the laboratory in Harare and be used to grow the gonorrhoea. This will be done right away. Tests will then be performed to check if the gonorrhoea is resistant to commonly used antibiotics. This is helpful for deciding what antibiotics should be recommended in guidelines.

For other tests to check resistance to antibiotics, the samples are stored in the laboratory in Zimbabwe during the study and tested at the end of the study. For some tests, including analysing the genes from the STIs, this may involve shipping your samples to a study laboratory outside Zimbabwe. If you are ok with this, an additional swab will be taken for these tests. Any of your samples sent to an external laboratory will be destroyed on completion of all the necessary tests. Destruction usually happens at the end of the study.

Importantly, you can participate in the study, but decline this part or other parts if you do not wish to undertake all of the procedures.

Finally, we will contact you by telephone after your baby is due to be born to find out information about your pregnancy, on partner notification, any thoughts on the testing processes, and to perform a repeat mental health screening. We will also check your health records and birth registers to gather additional information such as birth weight and delivery type.

RISKS AND DISCOMFORTS

Some of the topics that we discuss may be personal, and may bring up memories or feelings that you find upsetting or difficult. You can contribute as you wish, and there is no obligation to answer any question that you do not want to. Refusing to take part in some or all the questionnaires will not affect any services that you receive in any way, and we are not going to tell anyone the answers to your questions or what we have talked about.

BENEFITS AND/OR COMPENSATION

Testing may reveal that you have an STI. STIs can sometimes lead to problems in pregnancy, or infection of the new-born baby. Therefore, by testing for STIs we can offer treatment to reduce the chances of this happening. Treatment will be offered for free for chlamydia, gonorrhoea, trichomoniasis, and syphilis. If diagnosed with hepatitis B, we will refer you to a specialist for further assessment, and try to arrange for a hepatitis B vaccine to be administered to your new-born baby, to prevent your baby from getting hepatitis B.

This project will hopefully benefit others by providing important information on strategies to test for STIs in pregnancy. We cannot and do not guarantee or promise that you will receive any benefits from this study.

ALTERNATIVES TO PARTICIPATION

If you decide not to participate in this study, you will still receive routine pregnancy care. This includes testing for HIV and syphilis. Tests for chlamydia, gonorrhoea, trichomoniasis, and hepatitis B are not available in routine care in Zimbabwe.

Additionally, if you have symptoms of an STI and decide not to participate, you will still receive treatment to cover the possible causes of your symptoms in routine care.

CONFIDENTIALITY

If you agree to take part in this study by signing this document, all information obtained will be stored using a study number (instead of your name), in safe paper and computer files. No one will be able to access the information about you except for the research team.

We require your telephone number so that we can contact you after the birth to collect more information. Additionally, we may need to contact you if you have any positive results, but are unable to receive them on the same day. Contact details, and any other identifiable information, will be kept separately and will only be accessible to the research team.

With your permission, the healthcare team who provide your care at the clinic will be informed of your STI test results, so that your clinic records can be updated.

Any information that is obtained in connection with this study that can be identified with you will remain confidential and will be disclosed only with your permission. Under some circumstances, the MRCZ may need to review patient records for compliance audits.

ADDITIONAL COSTS

We hope to give you all of your results at the clinic on the same day as testing. If we are unable to do this, and you test positive for an STI, we will advise you to return to the clinic for treatment. Additionally, if we grow gonorrhoea in the lab that is resistant to the antibiotic we gave you, we will advise you to return to the clinic. We will cover the costs of treatment, but we will not be able to cover transport costs to reach the clinic.

DATA SHARING

Anonymised data from this study may be shared via a public data repository or by sharing directly with other researchers. You will not be identifiable by this information.

VOLUNTARY PARTICIPATION

Participation in this study is voluntary. If you decide not to participate in this study, your decision will not affect your future relations with any hospitals, clinics or other health services, nor with any health personnel or with the Biomedical Research and Training Institute or with the London School of Hygiene and Tropical Medicine. You are free to withdraw your consent and stop your involvement at any time without penalty.

IRB No. MRCZ/A/2899

SIGNATURE PAGE

Investigating point-of-care diagnostics for sexually transmitted infections and antimicrobial resistance in primary care in Zimbabwe (IPSAZ)

STI TESTING

Protocol Version 1.2 dated 10 May 2022

OFFER TO ANSWER QUESTIONS

Before you sign this form, please ask any questions on any aspect of this study that is unclear to you. You may take as much time as necessary to think it over.

AUTHORIZATION

You are making a decision whether or not to participate in this study. Your signature indicates that you have read and understood the information provided above, have had all your questions answered, and have decided to take part.

- I have read the information concerning this study and I understand what will be required
- I understand that at any time I can withdraw from this study without giving a reason
- I understand that data about/from me may be shared via a public data repository or by sharing directly with other researchers, and that I will not be identifiable from this information.

- I agree to have samples from my vagina or cervix, and any gonorrhoea grown from my samples, stored, and shipped to a laboratory outside Zimbabwe for tests mentioned in this consent form (*Mark either "Yes" or "No" with your initials to indicate your choice*)

Yes ☐ **No** ☐

____________________	____________________	☐☐/☐/20☐☐
Name of Participant (print)	*Signature of Participant*	*Date*
____________________	____________________	☐☐/☐/20☐☐
Name of Staff obtaining consent	*Signature of Staff*	*Date*
____________________	____________________	☐☐/☐/20☐☐
Name of Witness (if required)	*Signature of Witness*	*Date*

YOU WILL BE OFFERED A COPY OF THIS CONSENT FORM TO KEEP.

If you have any questions concerning this study or consent form beyond those answered by the investigator, including questions about the research, your rights as a research participant or research-related injuries; or if you feel that you have been treated unfairly and would like to talk to someone other than a member of the research team, please feel free to contact the Medical Research Council of Zimbabwe (MRCZ) on telephone (04)791792 or (04) 791193 and cell phone lines 0784 956 128. The MRCZ Offices are located at 20 Cambridge Road, Avondale, Harare. This consent form has been reviewed and approved by the MRCZ, the Biomedical Research and Training Institute Institutional Review Board, and the London School of Hygiene & Tropical Medicine Interventions Research Ethics Committee.

MEDICAL RESEARCH COUNCIL OF ZIMBABWE
10 DEC 2022
APPROVED
20 CAMBRIDGE ROAD, AVONDALE, HARARE
