# Supplementary material for: Investigating point-of-care diagnostics for sexually transmitted infections and antimicrobial resistance in antenatal care in Zimbabwe (IPSAZ): protocol for a mixed-methods study
Source: BMJ Open. 2023 Apr 20;13(4):e070889. doi: 10.1136/bmjopen-2022-070889 (PMC10124298; doi:10.1136/bmjopen-2022-070889)
Supplement: Author's manuscript [file bmjopen-2022-070889.draft_revisions.pdf]

BMJ Open

BMJ Open is committed to open peer review. As part of this commitment we make the peer review history of every article we publish publicly available.

When an article is published we post the peer reviewers' comments and the authors' responses online. We also post the versions of the paper that were used during peer review. These are the versions that the peer review comments apply to.

The versions of the paper that follow are the versions that were submitted during the peer review process. They are not the versions of record or the final published versions. They should not be cited or distributed as the published version of this manuscript.

BMJ Open is an open access journal and the full, final, typeset and author-corrected version of record of the manuscript is available on our site with no access controls, subscription charges or pay-per-view fees (http://bmjopen.bmj.com).

If you have any questions on BMJ Open's open peer review process please email info.bmjopen@bmj.com

BMJ Open

Investigating point-of-care diagnostics for sexually transmitted infections and antimicrobial resistance in antenatal care in Zimbabwe (IPSAZ): protocol for a mixed methods study

Journal:	*BMJ Open*
Manuscript ID	bmjopen-2022-070889
Article Type:	Protocol
Date Submitted by the Author:	08-Dec-2022
Complete List of Authors:	Martin, Kevin ; London School of Hygiene & Tropical Medicine, Clinical Research Department; Biomedical Research and Training Institute<br>Dziva Chikwari, Chido; London School of Hygiene & Tropical Medicine, Department of Infectious Disease Epidemiology; Biomedical Research and Training Institute<br>Dauya, E; Biomedical Research and Training Institute<br>Mackworth-Young, Constance; London School of Hygiene and Tropical Medicine Faculty of Public Health and Policy, Department of Global Health and Development; Biomedical Research and Training Institute<br>Bath, David ; London School of Hygiene and Tropical Medicine, Department of Global Health and Development<br>Tucker, Joseph; London School of Hygiene & Tropical Medicine, Clinical Research Department<br>Simms, Victoria; London School of Hygiene and Tropical Medicine, Department of Infectious Disease Epidemiology<br>Bandason, T; Biomedical Research and Training Institute<br>Ndowa, Francis; Skin & Genito-Urinary Medicine Clinic<br>Mugurungi, Owen; Ministry of Health and Child Care, AIDS and TB Unit<br>Machiha, Anna; Ministry of Health and Child Care, AIDS and TB Unit<br>Marks, Michael; London School of Hygiene & Tropical Medicine, Clinical Research Department; Hospital for Tropical Diseases,<br>Kranzer, Katharina; London School of Hygiene & Tropical Medicine, Clinical Research Department; Biomedical Research and Training Institute<br>Ferrand, Rashida; London School of Hygiene & Tropical Medicine, Department of Clinical Research
Keywords:	GENITOURINARY MEDICINE, HEALTH ECONOMICS, HIV & AIDS < INFECTIOUS DISEASES, Public health < INFECTIOUS DISEASES, MICROBIOLOGY, OBSTETRICS

SCHOLARONE™
Manuscripts

Investigating point-of-care diagnostics for sexually transmitted infections and antimicrobial resistance in antenatal care in Zimbabwe (IPSAZ): protocol for a mixed methods study

Kevin Martin[1 2 3], Chido Dziva Chikwari[2 4], Ethel Dauya[2], Constance Mackworth-Young[2 5], David Bath[5], Joseph D. Tucker[1], Victoria Simms[2 4], Tsitsi Bandason[2], Francis Ndowa,[6] Owen Mugurungi[7], Anna Machiha[7], Michael Marks[1 8 9], Katharina Kranzer[1 2 10], Rashida A. Ferrand[1 2]

1. Department of Clinical Research, London School of Hygiene & Tropical Medicine, London, UK

2. Biomedical Research and Training Institute, Harare, Zimbabwe

3. Department of Global Health and Infection, Brighton and Sussex Medical School, Brighton, UK

4. Department of Infectious Disease Epidemiology, London School of Hygiene & Tropical Medicine, London, UK

5. Department of Global Health and Development, London School of Hygiene & Tropical Medicine, London, UK

6. Skin & Genito-Urinary Medicine Clinic, Harare, Zimbabwe

7. AIDS and TB unit, Ministry of Health and Child Care, Harare, Zimbabwe

8. Hospital for Tropical Diseases, University College London Hospital, London, United Kingdom

9. Division of Infection and Immunity, University College London, London, United Kingdom

10. Division of Infectious and Tropical Medicine, Medical Centre of the University of Munich, Munich, Germany

Corresponding author
Dr Kevin Martin
Department of Clinical Research
London School of Hygiene & Tropical Medicine
London WC1E 7HT, United Kingdom
kevin.martin@lshtm.ac.uk

Word counts: Manuscript = 3906. Abstract = 291

For peer review only

Abstract

Introduction

Sexually transmitted infections (STIs) can cause serious morbidity, including pelvic inflammatory disease, and adverse pregnancy outcomes. In low- and middle-income countries, limited laboratory infrastructure has resulted in a syndrome-based approach being used for management of STIs, which has poor sensitivity and specificity, leading to considerable underdiagnosis and overtreatment. The World Health Organization (WHO) has called for development and evaluation of strategies to inform replacement of syndromic management by diagnostic testing.

The aim of this project is to evaluate a strategy of point-of-care testing for six STIs in antenatal care (ANC) in Zimbabwe.

Methods and analysis

A prospective interventional study will be conducted in ANC clinics in Harare Province, Zimbabwe. One thousand pregnant women will be recruited when registering for routine antenatal care and offered an integrated screening package including testing for *Chlamydia trachomatis* (CT), *Neisseria gonorrhoeae* (NG), *Trichomonas vaginalis* (TV), Hepatitis B, syphilis and HIV. All individuals with STIs will receive treatment, partner notification services, risk reduction counselling, and referral if needed according to national guidelines. Gonorrhoea samples will be cultured and tested for antimicrobial resistance as per WHO enhanced gonococcal antimicrobial surveillance programme guidelines.

The primary outcome measure is the composite prevalence of CT, NG, TV, syphilis and hepatitis B. A mixed methods process evaluation and economic evaluation will be conducted to understand the acceptability, feasibility, and cost-effectiveness of integrated STI testing, compared to standard of care (syndromic management).

Ethics and Dissemination

The study protocol was approved by the Medical Research Council of Zimbabwe, the Biomedical Research and Training Institute Institutional Review Board, and the London School of Hygiene & Tropical Medicine Research Ethics Committee. Results will be submitted to open-access peer-reviewed journals, presented at academic meetings and shared with participating communities and with national and international policy-making bodies.

For peer review only

Registration

https://clinicaltrials.gov/ct2/show/NCT05541081

Key words

Sexually transmitted infections; point-of-care testing; antenatal care; pregnancy; Zimbabwe

Article summary

Strengths and limitations of this study

- The mixed methods approach, with qualitative and quantitative data, will allow for the development of a multi-layered understanding of the acceptability and feasibility of this integrated screening package.
- Inclusion of an economic evaluation will allow for estimation of the cost-effectiveness of this screening package compared with routine care, which is essential for considering the scalability and sustainability of the programme.
- The large sample size will ensure an estimation of STI prevalence with high precision amongst antenatal attendees in Harare.
- This study does not include a formal outcome evaluation to assess the impact of this integrated screening package for STIs on adverse birth outcomes, which will need to be informed by future studies.
- Our focus on urban clinics will likely limit the generalisability of our findings to other urban centres in Southern Africa.

For peer review only

Introduction

Globally, there were an estimated 376 million infections of *Chlamydia trachomatis* (CT), *Neisseria gonorrhoeae* (NG), *Trichomonas vaginalis* (TV) and syphilis in 2016 among people aged 15 to 49 years.[1] Untreated, sexually transmitted infections (STIs) can cause adverse pregnancy outcomes, congenital infection and pelvic inflammatory disease.[1] STIs are also associated with increased risk of both HIV transmission and acquisiton.[2]

There are multiple contributing factors to the persistence of the high incidence of STIs globally. Complex sociocultural barriers such as stigma, limited sexual health education and barriers to condom use, and biomedical factors such as asymptomatic infections and increasing levels of antimicrobial resistance (AMR), have been exacerbated in recent years by a substandard global response to STIs, characterised by a lack of funding and political commitment.[3]

Effective management of STIs in low- and middle-income countries (LMIC) is additionally hindered by the use of syndromic management, which is the provision of treatment to an individual presenting with symptoms and/or signs that may be caused by an STI.[4] This is problematic as the majority of curable STIs are asymptomatic and are missed by syndromic management.[5] Furthermore, treatment for infections that patients may not have may lead to side effects and increased AMR.[6]

Diagnostic platforms that do not require complicated laboratory infrastructure are available, but cost and lack of evidence on how they should be implemented to maximise both clinical effectiveness and cost-effectiveness in LMICs limit their implementation. Integration of diagnostic STI testing into health systems is likely to be key to reducing rates of STIs in LMICs and the World Health Organization (WHO) has called for evidence to inform replacement of syndromic management by diagnostic testing.[7,8]

A group that may particularly benefit from the introduction of diagnostic testing for STIs is pregnant women. Pregnant women in LMICs are a high-risk population for STIs[9], and diagnosis and treatment may prevent adverse pregnancy outcomes and congenital transmission of some infections.[10] The integration of point-of-care (POC) testing for HIV and syphilis into ANC services provides a platform for further STI testing and management and potentially enhances operational feasibility. In addition to curable STIs, this is also pertinent for hepatitis B, as testing is noted to be essential for the WHO's triple elimination initiative, which aims to eliminate vertical transmission of HIV, syphilis, and hepatitis B.[11] Key to triple elimination is the provision of a multi-disciplinary approach within routine ANC services.

For peer review only

There is limited data on STI prevalence amongst pregnant women in Zimbabwe. Prevalence of CT up to 26.0%, of NG up to 6.4%, and TV up to 24.8% have been reported in pregnant women in South Africa and Zambia.[12-16] A 2010 study found a TV prevalence of 11.8% and syphilis prevalence of 1.2% among pregnant women in Harare.[17] Recent studies amongst female youth in Harare have demonstrated a combined CT/NG prevalence between 18.2% and 19.5%. [18,19] A prevalence of hepatitis B ranging between 3.1-5.3% has been reported in pregnant women in South Africa [20-22].

Control of STIs, particularly NG, is additionally compromised by AMR.[23] Surveillance is key to identifying and monitoring AMR. WHO and the Centers for Disease Control, USA established the Enhanced Global Gonococcal Antimicrobial Surveillance Programme (EGASP) in 2015.[24] A sample of at least 100 gonococcal isolates per year per country is recommended.[23] However, gonococcal AMR data is still extremely limited and in 2018, only five of forty-seven countries in the WHO African Region reported susceptibility testing for NG of at least one of ceftriaxone, cefixime, ciprofloxacin and azithromycin.[25-28]

Rationale

Evidence is required to inform the use of diagnostic testing for STIs in LMICs, at both national and international levels, particularly regarding acceptability, feasibility, and cost-effectiveness. Additionally, there is a data gap regarding the prevalence of STIs amongst pregnant women in Zimbabwe. Given the paucity of data on AMR in NG in Africa, there is also a need to strengthen AMR surveillance systems.

Aims and objectives

The overall aim of this study is to implement and evaluate a strategy for integration of POC diagnostics for STIs into ANC settings and to establish a gonococcal antimicrobial resistance surveillance strategy aligned with EGASP in Zimbabwe.

The objectives are to:

1. Determine the prevalence and yield of point-of-care testing for CT, NG, TV, syphilis and hepatitis B, and factors associated with presence of STIs, among pregnant women

2. Conduct a mixed-methods process evaluation to understand the acceptability and feasibility of POC STI testing and comprehensive case management in ANC settings

3. Estimate the cost and cost-effectiveness of integrated STI testing compared to standard of care

For peer review only

4. Investigate the prevalence of AMR for NG to inform the development of an EGASP in Zimbabwe

Methods and analysis

Study design and setting

A prospective interventional study will be conducted in primary healthcare clinics (PHCs) in Harare province, Zimbabwe. The PHCs are all based in urban, high-density settings, and provide nurse-led services including antenatal care and uncomplicated deliveries. High-risk women receive their ANC at central hospitals, with referral also available if complications develop in labour. Opt-out HIV and syphilis testing using rapid diagnostic tests is part of routine care. GeneXpert devices are often available for tuberculosis diagnosis, but the study will provide an additional machine to ensure that sufficient diagnostic capacity is available. We previously demonstrated the feasibility of using non-laboratory technicians to operate the GeneXpert device for on-site CT/NG testing in community settings in Bulawayo, Zimbabwe.[29]

Study population and recruitment

Pregnant women will be recruited when registering for routine antenatal care, starting in January 2023. It is the intention that only pregnant women attending their first antenatal care visit of this pregnancy will be recruited. However, if there is ongoing slower than expected recruitment, pregnant women attending for antenatal care follow-up visits will also be considered for enrolment. There will be no age cut-off for enrolment. Exclusion criteria will be enrolment in this study on a previous antenatal visit and being unable or unwilling to provide written informed consent. Recruitment will be conducted during weekdays only. Pregnant women will be consecutively enrolled as testing capacity allows.

Reasons for declining participation, and for exclusion, will be documented. If participants only consent to some of the STI tests, reasons for declining the others will be recorded.

Study procedures

The full schedule of events for pregnant women is described in Table 1. Following consent, an interviewer-administered questionnaire will collect sociodemographic data, clinical history including STI symptoms and recent antibiotic use, sexual and obstetric history. Contact information will be collected for follow-up.

For peer review only

Table 1: Schedule of events for pregnant women

Participants	Activity	Day 0 **(Study entry)**	Day 1-5	Day 4-14	On partner attendance	Birth	Post-partum **(planned telephone follow-up)**
All	Informed consent	X					
All	Questionnaire	X					
All	HIV testing	X					
All	Syphilis testing	X					
All	HBV testing	X					
All	CT/NG testing	X					
All	TV testing	X					
STI test +ve	Health education	X					
HBV test +ve	Venepuncture	X					
HBV test +ve	HBV viral load and ALT testing	X					
HBV test +ve	Referral to secondary care	X					
HIV test +ve	Referral as per PHC processes	X					
CT/NG/TV/syphilis test +ve	Provision of treatment	X					
CT/NG/TV/syphilis test +ve	Partner notification advice and slip	X					
NG test +ve	Cervical swab collection	X					
NG test +ve	Plating of cervical swab & incubation at laboratory	X					
STI test +ve<br>Not treated on day 0	Contact participant by telephone and ask to return to PHC for treatment		X				
Cultured NG isolate	Storage of isolate at -80°C			X			
Partners	Provision of treatment to partners that attend PHC				X		

For peer review only

HBV test +ve	Provision of HBV birth dose vaccine co-ordinated with PHC and secondary care					X	
CT/NG/TV/syphilis test +ve	Contact by telephone to collect data on partner notification process						X
All	Contact by telephone to collect birth outcome data						X
All	Review of birth registry records to supplement birth outcome data from participants						X

ALT = Alanine aminotransferase. CT = Chlamydia. HBV = Hepatitis B virus. HIV = Human immunodeficiency virus. NG = Gonorrhoea. PHC = Primary healthcare centre. RDT = Rapid diagnostic test. STI = Sexually transmitted infection. TV = Trichomoniasis.

For peer review only

Participants will provide three self- or provider-taken vaginal swab samples. One vaginal swab sample will be tested for CT and NG using the Xpert® CT/NG assay (Cepheid), which has an analytic time of 90 minutes. The GeneXpert device will be operated using a rechargeable powerpack to provide an uninterrupted power supply. The second swab will be tested for TV using the OSOM® Trichomonas Rapid Test (Sekisui Diagnostics), which has an analytic time of 10 minutes.[30] The third swab will be stored for future studies including possible whole genome sequencing. A fingerprick blood sample will be taken for HIV, syphilis and hepatitis B testing using the SD BIOLINE HIV/Syphilis Duo (Abbott Diagnostics Medical Co. Ltd) (analytic time 20 minutes) and HBsAg 2 (Abbott Diagnostics Medical Co. Ltd) (analytic time 30 minutes) rapid tests, respectively.[31] HIV and syphilis testing, referral to HIV services, syphilis treatment, and partner notification for those with HIV or syphilis, are already part of routine care. The study team will work with health facility staff to integrate the additional STI testing with routine ANC services to prevent duplication of procedures.

Participants with positive test results and their partners will be managed in line with Zimbabwe national treatment guidelines.[32] For participants with an STI syndrome on presentation, immediate treatment will be provided for syndromes such as pelvic inflammatory disease and genital ulcer disease, where testing will not alter management. For vaginal discharge syndrome, participants will ideally wait for their results to receive tailored treatment, however they will receive metronidazole regardless of results, in order to cover for bacterial vaginosis. For symptomatic participants not willing or able to wait or return for their results, they will have the option to receive full syndromic treatment.

Participants will ideally collect their results and receive treatment if necessary within the same clinical visit. Participants who test positive for an STI but are unable to receive same day treatment, will be actively followed up by telephone, up to five times over a 28 day period, to advise them to return for treatment.

A client-referral approach will be used for notification of sexual partners. Women will be counselled on the importance of their partners receiving treatment and given partner notification (PN) slips for their partners to return for presumptive treatment. Although partners will be able to attend any clinic, treatment will be provided free-of-charge if they return to the study clinic.

Women newly diagnosed with HIV will be referred for antiretroviral therapy as per local PHC processes. Women newly diagnosed with hepatitis B will have hepatitis B viral load and alanine transaminase (ALT) testing, alongside referral to a gastroenterology specialist in secondary care. Although hepatitis B vaccination is currently included in the Zimbabwe national vaccination schedule at six, ten, and fourteen weeks, birth dose vaccination is not yet standard of care in Zimbabwe. Birth

For peer review only

dose vaccines will therefore be provided by the IPSAZ study. This is likely to be logistically complex and bespoke strategies for implementation will be designed in conjunction with healthcare teams at the individual PHCs and with local secondary care providers.

Birth outcome data will be collected from birth registers, including birthweight, gestation, mode of delivery, and still birth. Participants will also be contacted by telephone post-partum to facilitate this process by providing information on date and location of birth, and to provide supplemental data if necessary if the birth register is incomplete. Participants will also be asked about number of ANC visits, and for women with positive STI results, if they gave the PN slip to their partner, and if their partners were treated.

Process evaluation

A mixed-methods process evaluation will be conducted, based on the MRC Process Evaluation Framework.[33] The focus will be on understanding what was implemented and how; how the intervention led to change; and how local context affects implementation and shapes outcomes. Steckler and Linnan's process evaluation framework has also guided the choice of specific research domains related to implementation, where fidelity, dose and reach/coverage, are central features.[34]

Table 2 details the process evaluation research domains and questions. A logic model demonstrating the proposed theory of change is shown in Figure 1.

For peer review only

Table 2: Framework for process evaluation.

Framework domain	Research domain	Research questions	Data collection methods and sources
Implementation	Fidelity	• How did implementation vary from the protocol i.e. (a) offering STI screening, (b) undertaking STI screening, (c) providing comprehensive case management including partner notification, (d) training and supervision of staff<br>• What were the barriers and facilitators to implementation?<br>• What adaptations were made?	Routine monitoring data<br>Structured observation<br>FGDs with clinic staff (1-2 per PHC) and IDIs with research team (all members), pregnant women (8-10 per PHC), partners (3-4 per PHC) and clinic staff (4 per PHC) *<br>IDIs with pregnant women who decline STI screening (3-4 per PHC) to explore reasons for this.
	Coverage	• What proportion of: (a) Pregnant women attending antenatal care were offered STI screening, (b) Pregnant women who were offered STI screening took it up (c) Positive STI cases were treated, (d) Partners of positive cases were treated?<br>• How equitable was this coverage?<br>• What were the barriers and facilitators to each step?	
Mechanisms of Impact	Responses to and interactions with the intervention	• Which components of the intervention were best accepted and adopted by pregnant women and HCWs and why?<br>• What challenges and barriers were faced?	Structured Observation<br>FGDs and IDIs with pregnant women, partners, and HCWs*
	Interactions and Consequences	• How did various components of the intervention interact?<br>• Were there any unanticipated pathways or consequences?	
Context	Proximal and distal	• What social, cultural, political, and logistical factors impede or facilitate how the intervention was implemented, and how were HCWs able to engage with and adopt aspects of the intervention?<br>• What were contextual reasons for adaptations to the intervention and its delivery?	FGDs and IDIs with pregnant women, partners, and HCWs*<br>Structured and unstructured observations<br>Key informant interviews with local health authorities, and community leaders<br>Context diaries to record external events

STI = Sexually transmitted infection. HCW = Healthcare worker. IDI = In-depth interview. FGD = Focus group discussion. PHC = Primary healthcare clinic.
*Number of IDIs/FGDs listed are approximations.

For peer review only

Following initial qualitative formative work to refine the testing strategy, a concurrent triangulation strategy will be used, with quantitative and qualitative data collected in parallel, with similar weighting given to each.[35] This will allow for triangulation of data in order to comprehensively address the process evaluation questions. Routine monitoring data will include uptake of testing, treatment, and partner notification, as well as stock monitoring, debriefing minutes, and recording of GeneXpert error codes. Data collection will also include structured and unstructured observation, and focus group discussions and in-depth interviews with key stakeholders including pregnant women, partners, clinic staff, policymakers, and the research team involved in delivery of the intervention (including both the clinic-based team and laboratory staff). Different topic guides will be designed for interviews and focus group discussions at different stages of implementation, to reflect the changing focus of the process evaluation. Participants for interviews and group discussions will be purposively selected to ensure a relevant range of views for each stage of the process evaluation. Adaptations will be made to the testing strategy based on interim process evaluation findings to improve ongoing implementation.

Thematic analysis on qualitative data will be performed to identify and develop key themes and concepts on addressing what was implemented and how; how the intervention led to change; and how local context affects implementation and shapes outcomes.

Economic evaluation

Cost-effectiveness will be evaluated as the ratio of incremental costs and incremental effects of point-of-care testing for CT, NG, and TV, in comparison with routine care using syndromic management. Total costs and effects will be estimated for each arm, with incremental values calculated from these estimates.

Clinical endpoints include mild infection, pelvic inflammatory disease, infertility, and adverse birth outcomes including miscarriage, stillbirth, low birthweight, and prematurity. Disability-adjusted life years will be the main outcome, and will be modelled based on clinical endpoints, predicted life expectancy and disability weights.[36] Intermediate measures include cost per patient screened, diagnosed with an STI, and treated. A simple static decision tree model will be used to structure the cost-effectiveness evaluation, which will be from a health system perspective.

Health system costs required to deliver the testing strategy will be collected from both primary and secondary data sources using a bespoke cost extraction tool. Financial and economic costs will be estimated based on study financial records, staff interviews, time-and-motion studies, and other sources such as national salary scales for the Zimbabwe health service. Costs categories will include

For peer review only - http://bmjopen.bmj.com/site/about/guidelines.xhtml

start-up costs such as training costs, capital costs including buildings and storage and equipment, and recurrent costs, including personnel, consumables, and transport costs. Costs associated with downstream complications of infection will be estimated from the literature. Costs incurred by patients whilst attending ANC, including transport costs and opportunity costs, will be collected using the interviewer-administered questionnaire. Additional costs associated with the testing strategy, such as a longer visit or to return for treatment, will be recorded.

Individual and combined parameter uncertainty will be investigated using deterministic and probabilistic sensitivity analyses. The cost-effectiveness of the testing strategy compared to syndromic management will be compared against appropriate cost-effectiveness thresholds for Zimbabwe.

Antimicrobial resistance surveillance

EGASP recommends the collection of at least 100 gonococcal isolates per year per country. Assuming a prevalence of NG of 3% amongst pregnant women, only 30 women with NG will be identified. To supplement the number of isolates obtained from ANC participants, men aged 18 years and above presenting with urethral discharge to the study clinics who provide written informed consent will be recruited. Consecutive sampling will be performed as resources allow, with the antenatal testing strategy taking priority.

Men with urethral discharge have been selected as the yield of a positive gonococcal culture will be high. Assuming an estimate of 50% NG prevalence amongst symptomatic men,[37] we anticipate recruiting approximately 140 males with urethral discharge. We therefore estimate isolation of 70 gonococcal isolates from men which, in addition to 30 isolates from women, will give 100 NG isolates in total. If the prevalence of NG is lower than predicted, recruitment of men with urethral discharge will continue until 100 NG isolates have been obtained.

Data on age, sex, risk factors for STIs including number of recent sexual partners and gender of sexual partners, current STI symptoms, recent antibiotic use, treatment provided at the PHC, and previous STI diagnoses including HIV, will be collected using an interview-administered questionnaire. Nurse-collected urethral swabs will be collected from all enrolled men, and they will be managed syndromically according to national guidelines as part of routine care.

For peer review only

Testing procedures for antimicrobial resistance

WHO EGASP guidelines and standard operating procedures will be adapted for use with pregnant women, and otherwise followed for AMR testing for NG.[38] Nurse-collected cervical samples will be obtained using ESwabs® (COPAN Diagnostics Inc.) from women initially found to be positive for NG using the Xpert assay. Samples will be plated onto the InTray® GC (Biomed Diagnostics Inc.) in-vitro device, as will urethral samples collected from men. These will be subsequently incubated in the laboratory and minimum inhibitory concentrations of ceftriaxone, cefixime, azithromycin and ciprofloxacin will be determined using Etest® (bioMérieux) and interpreted using the European Committee on Antimicrobial Susceptibility Testing (EUCAST) standards.[39]

Sample size calculations

There is limited data on STI prevalence in pregnant women in Zimbabwe. A prevalence of curable STIs between 32.0% and 37.0% has been reported in South Africa and Zambia.[12-16] Our recent studies among female youth in Harare have demonstrated combined CT/NG prevalence between 18.2% and 19.5%. [18,19] The prevalence of hepatitis B in pregnant women in South Africa has ranged between 3.1% and 5.3%.[20-22] Therefore, a conservative estimate of composite prevalence of curable STIs and hepatitis B is 30.0%. With a desired precision of 3% and alpha of 0.05, a sample size of 896 is required. To allow for invalid test results, 1000 pregnant women will be screened. The minimum detectable odds ratios for factors associated with the presence of STIs at different composite prevalence of STIs are shown in Table 3.

Table 3: Minimum detectable odds ratios for factors associated with presence of STIs

Sample size*	STI prevalence	Precision	Smallest OR detected at 80% power	Smallest OR detected at 90% power
896	20%	2.6%	1.74	1.89
	25%	2.8%	1.67	1.80
	30%	3.0%	1.63	1.75

*Assuming a prevalence of a risk factor of 20% amongst those without STIs. OR = Odds ratio. STI = Sexually transmitted infection.

Statistical analysis

The primary outcome measure is the composite prevalence of CT, NG, TV, syphilis and hepatitis B in this population.

Other outcome measures include:

1) Individual prevalence of each STI

For peer review only

2) Uptake of testing

3) Uptake of treatment

4) Uptake of partner notification

5) STI yield (number of participants with a positive STI result/total number of eligible individuals)

6) Prevalence of antimicrobial resistance to ceftriaxone, cefixime, azithromycin and ciprofloxacin in NG isolates

7) Prevalence of preterm birth, miscarriage, and low birth weight

Categorical variables will be described using frequencies and percentages. Continuous variables will be described using either mean (standard deviation) or median (interquartile range) for normally distributed and non-normally distributed data, respectively. Multivariable logistic regression will be used to assess factors associated with presence of STIs. Clustering will be adjusted for at clinic level. Logistic regression will also be used to assess the relationship between STI diagnosis and birth outcomes.

Data management procedures

Quantitative data will be collected using electronic case report forms on tablet computers using Open Data Kit software, with range restrictions and dropdown menus too minimise data entry errors. CT/NG results recorded on tablet computers will be cross-checked with the readout from the GeneXpert device. Data will be managed and cleaned using STATA (StataCorp, Texas, USA). Interviews and focus group discussions will be audio recorded, and transcribed verbatim. Anonymisation of transcripts will be performed once translation and transcription has been completed.

All data will be stored in password-controlled databases, and all data will be encrypted. All data collected will be anonymised using a unique study ID. Any identifiable data (e.g., locator forms and consent forms) will be stored in secure, locked facilities with access limited to the study team.

Data sharing

The informed consent procedure will clarify the sharing of anonymised data, either via a public data repository, or by directly sharing with other researchers.

At the time of publication of research, the subset of data required for the purposes of verifying research findings will be made available for sharing and will be placed in Data Compass (the LSHTM institutional research data repository – accessible at https://datacompass.lshtm.ac.uk/). This repository

For peer review only

will enable direct download of records with codebooks to enable replication of the data analyses. A more complete sharing of data with any research group requesting access to individual data records will be done 12 months after publication. At this point, all data and study tools will be made available through Data Compass. Data for sharing will be de-identified prior to release. Details of how to access data will be published with each study publication.

Patient and public involvement

Initial formative work will include dialogue and input from service users to help refine the testing strategy and data collection tools. Findings will also be disseminated through study clinics.

Ethics and dissemination

The study protocol has been approved by the Medical Research Council of Zimbabwe (MRCZ/A/2899), the Biomedical Research and Training Institute Institutional Review Board (AP176/2022) and the London School of Hygiene & Tropical Medicine Research Ethics Committee (26787).

Written informed consent to participate in the study will be obtained in the preferred language of the potential participant (English or Shona). Specific consent will also be sought for storage of samples and the sharing of anonymised data via a public data repository. Participants will not be identifiable from this information.

In Zimbabwe, individuals who are under 18 years of age and pregnant, are considered emancipated minors. Therefore, independent informed consent will be obtained from pregnant minors.

For any participants who are under 16 years of age and pregnant, we will consider on an individual basis whether further input is needed regarding child protection. This may include discussion of the case with a multidisciplinary team and possible referral to social services. If these instances do arise, we aim to integrate into existing clinic processes as much as possible.

Results will be submitted to open-access peer-reviewed journals, presented at academic meetings and shared with participating communities and with national and international policy-making bodies.

For peer review only

Discussion

There is mounting evidence that syndromic management is not an effective method for the control of STIs.[14,40,41] However, evidence is required by national and international policymakers to inform how to transition towards diagnostic testing for STIs. We anticipate high prevalence of STIs among pregnant women in this study, thus providing additional evidence that new strategies are required for control of STIs in Zimbabwe. The data on acceptability, feasibility, and cost-effectiveness, will provide guidance on how best to implement new strategies for a package of integrated diagnostic testing in antenatal care. Furthermore, this study will provide baseline data for the design of future testing strategies, implemented at a larger scale, and potentially as part of a cluster-randomised trial to demonstrate efficacy.

The IPSAZ study has several strengths. The large sample size ensures that STI prevalence in the antenatal population in Harare will be estimated with high precision. The use of mixed methods for the process evaluation will enable quantitative and qualitative data to complement and inform each other. Furthermore, the economic evaluation will inform policymakers about whether introducing such a testing programme represents a cost-effective use of health resources in this and similar contexts. Finally, we will be following adapted EGASP standard procedures for gonococcal culture and AMR testing. EGASP guidelines do not currently include provision for sample collection from pregnant women, and are focussed on symptomatic men. Our experience will therefore inform the potential expansion of EGASP guidelines to include pregnant women, and to consider the use of antenatal networks as a platform for gonococcal surveillance for future national EGASP programmes.

We acknowledge some limitations. There is no formal outcome evaluation comparing birth outcomes between those who received the intervention, and those who did not. This is an important research question. Although treatment of STIs is likely to prevent complications such as pelvic inflammatory disease, there is conflicting evidence on whether provision of treatment for STIs during pregnancy prevents adverse birth outcomes.[42-44] Future studies need to investigate the clinical effectiveness of scaling-up of STI testing and treatment in antenatal care.

The testing strategy will be delivered by members of a dedicated study team, which may not be representative of how testing would integrate into existing governmental clinical services, especially given that resources and staff are severely constrained in Zimbabwe. However, the mixed methods approach to the process evaluation will provide supporting information to contextualise the findings.

Finally, the study will be conducted in urban PHCs in Harare province. As a result, the results will likely be generalisable to urban centres in Southern Africa, but less so to rural areas in this region.

For peer review only

In summary, the IPSAZ study will provide important data to develop an integrated screening package for STIs in antenatal care in Southern Africa. The epidemiological data, process evaluation, and economic evaluation will all help inform sustainability and scalability, in order to provide evidence-based policy recommendations. Additionally, the collection of gonococcal AMR data will also be used to inform national STI treatment guidelines in Zimbabwe.

Data availability statement

No data is associated with this paper.

Author contributions

KM conceptualised the IPSAZ study, supervised by RAF, KK, and MM. KM developed the first draft of the paper, developed the study manual of operations, and will be responsible for study implementation. KM and TB are responsible for data management. CDC, ED, CMY, DB, JT, VS, TB, FN, OM, and AM, provided guidance on study design and methodology. All authors reviewed the final draft of the manuscript.

Funding statement

This work was supported by the Wellcome Trust grant number 225468/Z/22/Z awarded to KM.

Competing interests

No competing interests were disclosed.

For peer review only

References

1. Rowley J, Vander Hoorn S, Korenromp E, et al. Chlamydia, gonorrhoea, trichomoniasis and syphilis: global prevalence and incidence estimates, 2016. *Bull World Health Organ* 2019; **97**(8): 548-62P.
2. Fleming DT, Wasserheit JN. From epidemiological synergy to public health policy and practice: the contribution of other sexually transmitted diseases to sexual transmission of HIV infection. *Sex Transm Infect* 1999; **75**(1): 3-17.
3. World Health Organization. Global health sector strategies on, respectively, HIV, viral hepatitis and sexually transmitted infections for the period 2022-2030, 2022.
4. World Health Organization. Guidelines for the management of symptomatic sexually transmitted infections, 2021.
5. Farley TA, Cohen DA, Elkins W. Asymptomatic sexually transmitted diseases: the case for screening. *Prev Med* 2003; **36**(4): 502-9.
6. Peeling RW, Holmes KK, Mabey D, Ronald A. Rapid tests for sexually transmitted infections (STIs): the way forward. *Sex Transm Infect* 2006; **82 Suppl 5**(Suppl 5): v1-6.
7. Toskin I, Blondeel K, Peeling RW, Deal C, Kiarie J. Advancing point of care diagnostics for the control and prevention of STIs: the way forward. *Sex Transm Infect* 2017; **93**(S4): S81-s8.
8. World Health Organization. Global Health Sector Strategy on sexually transmitted infections 2016-2021, 2016.
9. Joseph Davey DL, Shull HI, Billings JD, Wang D, Adachi K, Klausner JD. Prevalence of Curable Sexually Transmitted Infections in Pregnant Women in Low- and Middle-Income Countries From 2010 to 2015: A Systematic Review. *Sex Transm Dis* 2016; **43**(7): 450-8.
10. Mullick S, Watson-Jones D, Beksinska M, Mabey D. Sexually transmitted infections in pregnancy: prevalence, impact on pregnancy outcomes, and approach to treatment in developing countries. *Sex Transm Infect* 2005; **81**(4): 294-302.
11. Cohn J, Owiredu MN, Taylor MM, et al. Eliminating mother-to-child transmission of human immunodeficiency virus, syphilis and hepatitis B in sub-Saharan Africa. *Bull World Health Organ* 2021; **99**(4): 287-95.
12. Moodley D, Moodley P, Sebitloane M, et al. High prevalence and incidence of asymptomatic sexually transmitted infections during pregnancy and postdelivery in KwaZulu Natal, South Africa. *Sex Transm Dis* 2015; **42**(1): 43-7.
13. Green H, Taleghani S, Nyemba D, Myer L, Davey DJ. Partner notification and treatment for sexually transmitted infections among pregnant women in Cape Town, South Africa. *Int J STD AIDS* 2020; **31**(13): 1282-90.
14. Nyemba DC, Medina-Marino A, Peters RPH, et al. Prevalence, incidence and associated risk factors of STIs during pregnancy in South Africa. *Sex Transm Infect* 2020.
15. Joseph Davey DL, Nyemba DC, Gomba Y, et al. Prevalence and correlates of sexually transmitted infections in pregnancy in HIV-infected and- uninfected women in Cape Town, South Africa. *PLoS One* 2019; **14**(7): e0218349.
16. Chaponda EB, Chico RM, Bruce J, et al. Malarial Infection and Curable Sexually Transmitted and Reproductive Tract Infections Among Pregnant Women in a Rural District of Zambia. *Am J Trop Med Hyg* 2016; **95**(5): 1069-76.
17. Kurewa NE, Mapingure MP, Munjoma MW, Chirenje MZ, Rusakaniko S, Stray-Pedersen B. The burden and risk factors of Sexually Transmitted Infections and Reproductive Tract Infections among pregnant women in Zimbabwe. *BMC Infectious Diseases* 2010; **10**(1): 127.
18. Martin K, Olaru ID, Buwu N, et al. Uptake of and factors associated with testing for sexually transmitted infections in community-based settings among youth in Zimbabwe: a mixed-methods study. *Lancet Child Adolesc Health* 2021.
19. Kranzer K, Simms V, Dauya E, et al. Identifying youth at high risk for sexually transmitted infections in community-based settings using a risk prediction tool: a validation study. *BMC Infectious Diseases* 2021; **21**(1): 1234.

For peer review only

20. Chotun N, Preiser W, van Rensburg CJ, et al. Point-of-care screening for hepatitis B virus infection in pregnant women at an antenatal clinic: A South African experience. *PLoS One* 2017; **12**(7): e0181267.
21. Andersson MI, Maponga TG, Ijaz S, et al. The epidemiology of hepatitis B virus infection in HIV-infected and HIV-uninfected pregnant women in the Western Cape, South Africa. *Vaccine* 2013; **31**(47): 5579-84.
22. Thumbiran NV, Moodley D, Parboosing R, Moodley P. Hepatitis B and HIV co-infection in pregnant women: indication for routine antenatal hepatitis B virus screening in a high HIV prevalence setting. *S Afr Med J* 2014; **104**(4): 307-9.
23. Wi T, Lahra MM, Ndowa F, et al. Antimicrobial resistance in Neisseria gonorrhoeae: Global surveillance and a call for international collaborative action. *PLoS Med* 2017; **14**(7): e1002344.
24. Weston EJ, Wi T, Papp J. Strengthening Global Surveillance for Antimicrobial Drug-Resistant Neisseria gonorrhoeae through the Enhanced Gonococcal Antimicrobial Surveillance Program. *Emerg Infect Dis* 2017; **23**(13): S47-52.
25. Latif AS, Gwanzura L, Machiha A, et al. Antimicrobial susceptibility in Neisseria gonorrhoeae isolates from five sentinel surveillance sites in Zimbabwe, 2015-2016. *Sex Transm Infect* 2018; **94**(1): 62-6.
26. Unemo M, Lahra MM, Cole M, et al. World Health Organization Global Gonococcal Antimicrobial Surveillance Program (WHO GASP): review of new data and evidence to inform international collaborative actions and research efforts. *Sex Health* 2019; **16**(5): 412-25.
27. Takuva S, Mugurungi O, Mutsvangwa J, et al. Etiology and antimicrobial susceptibility of pathogens responsible for urethral discharge among men in Harare, Zimbabwe. *Sex Transm Dis* 2014; **41**(12): 713-7.
28. WHO Global Health Observatory. WHO Gonococcal AMR Surveillance Programme. https://www.who.int/data/gho/data/themes/topics/who-gonococcal-amr-surveillance-programme-who-gasp (accessed 17 Dec 2020).
29. Martin K, Dziva Chikwari C, Mackworth-Young CRS, et al. "It was difficult to offer same day results": evaluation of community-based point-of-care testing for sexually transmitted infections among youth using the GeneXpert platform in Zimbabwe. *BMC Health Services Research* 2022; **22**(1): 171.
30. Gaydos CA, Klausner JD, Pai NP, Kelly H, Coltart C, Peeling RW. Rapid and point-of-care tests for the diagnosis of Trichomonas vaginalis in women and men. *Sex Transm Infect* 2017; **93**(S4): S31-s5.
31. World Health Organization. Public reports of WHO prequalified IVDs. https://www.who.int/diagnostics_laboratory/evaluations/pq-list/hbsag/public_report/en/ (accessed Dec 17 2020).
32. Zimbabwe Ministry of Health and Child Care. National STI Management Guidelines. 2019.
33. Moore GF, Audrey S, Barker M, et al. Process evaluation of complex interventions: Medical Research Council guidance. *BMJ : British Medical Journal* 2015; **350**: h1258.
34. Linnan L, Steckler A. Process evaluation for public health interventions and research. San Francisco: Jossey-Bass; 2002.
35. Creswell JW. Research Design: Qualitative, Quantitative, and Mixed Methods Approaches: Sage; 2011.
36. GBD 2019 Diseases and Injuries Collaborators. Global burden of 369 diseases and injuries in 204 countries and territories, 1990-2019: a systematic analysis for the Global Burden of Disease Study 2019. *Lancet* 2020; **396**(10258): 1204-22.
37. Rietmeijer CA, Mungati M, Machiha A, et al. The Etiology of Male Urethral Discharge in Zimbabwe: Results from the Zimbabwe STI Etiology Study. *Sex Transm Dis* 2018; **45**(1): 56-60.
38. World Health Organization. Enhanced Gonococcal Antimicrobial Surveillance Programme (EGASP): General Protocol, 2021.
39. The European Committee on Antimicrobial Susceptibility Testing. Breakpoint tables for interpretation of MICs and zone diameters. Version 8.0. 2022. https://www.eucast.org/fileadmin/src/media/PDFs/EUCAST_files/Breakpoint_tables/v_12.0_Breakpoint_Tables.pdf.

40. Garrett NJ, Osman F, Maharaj B, et al. Beyond syndromic management: Opportunities for diagnosis-based treatment of sexually transmitted infections in low- and middle-income countries. *PLoS ONE* 2018; **13**(4): e0196209.
41. Verwijs MC, Agaba SK, Sumanyi JC, et al. Targeted point-of-care testing compared with syndromic management of urogenital infections in women (WISH): a cross-sectional screening and diagnostic accuracy study. *Lancet Infect Dis* 2019; **19**(6): 658-69.
42. Ravindran J, Richardson BA, Kinuthia J, et al. Chlamydia, Gonorrhea, and Incident HIV Infection During Pregnancy Predict Preterm Birth Despite Treatment. *J Infect Dis* 2021; **224**(12): 2085-93.
43. Folger AT. Maternal Chlamydia trachomatis infections and preterm birth:the impact of early detection and eradication during pregnancy. *Matern Child Health J* 2014; **18**(8): 1795-802.
44. Liu B, Roberts CL, Clarke M, Jorm L, Hunt J, Ward J. Chlamydia and gonorrhoea infections and the risk of adverse obstetric outcomes: a retrospective cohort study. *Sex Transm Infect* 2013; **89**(8): 672-8.

For peer review only

Figure 1: Logic model for mixed methods process evaluation

For peer review only

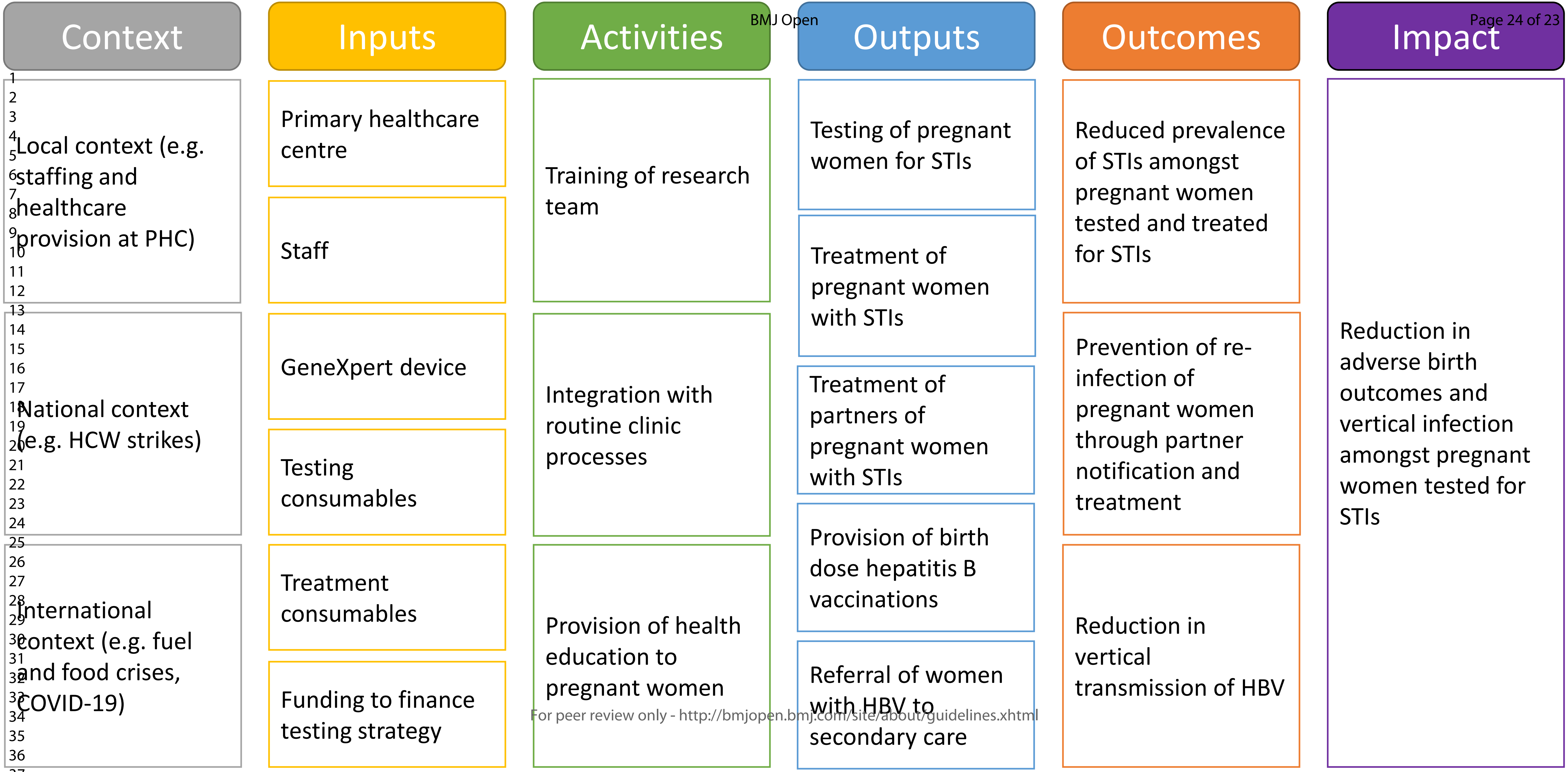
Context
Local context (e.g. staffing and healthcare provision at PHC)
National context (e.g. HCW strikes)
International context (e.g. fuel and food crises, COVID-19)
Inputs
Primary healthcare centre
Staff
GeneXpert device
Testing consumables
Treatment consumables
Funding to finance testing strategy
Activities
Training of research team
Integration with routine clinic processes
Provision of health education to pregnant women
Outputs
Testing of pregnant women for STIs
Treatment of pregnant women with STIs
Treatment of partners of pregnant women with STIs
Provision of birth dose hepatitis B vaccinations
Referral of women with HBV to secondary care
Outcomes
Reduced prevalence of STIs amongst pregnant women tested and treated for STIs
Prevention of re-infection of pregnant women through partner notification and treatment
Reduction in vertical transmission of HBV
Impact
Reduction in adverse birth outcomes and vertical infection amongst pregnant women tested for STIs

Investigating point-of-care diagnostics for sexually transmitted infections and antimicrobial resistance in antenatal care in Zimbabwe (IPSAZ): protocol for a mixed methods study

Journal:	*BMJ Open*
Manuscript ID	bmjopen-2022-070889.R1
Article Type:	Protocol
Date Submitted by the Author:	09-Mar-2023
Complete List of Authors:	Martin, Kevin ; London School of Hygiene & Tropical Medicine, Clinical Research Department; Biomedical Research and Training Institute<br>Dziva Chikwari, Chido; London School of Hygiene & Tropical Medicine, Department of Infectious Disease Epidemiology; Biomedical Research and Training Institute<br>Dauya, E; Biomedical Research and Training Institute<br>Mackworth-Young, Constance; London School of Hygiene and Tropical Medicine Faculty of Public Health and Policy, Department of Global Health and Development; Biomedical Research and Training Institute<br>Bath, David ; London School of Hygiene and Tropical Medicine, Department of Global Health and Development<br>Tucker, Joseph; London School of Hygiene & Tropical Medicine, Clinical Research Department<br>Simms, Victoria; London School of Hygiene and Tropical Medicine, Department of Infectious Disease Epidemiology<br>Bandason, T; Biomedical Research and Training Institute<br>Ndowa, Francis; Skin & Genito-Urinary Medicine Clinic<br>Katsidzira, Leolin; University of Zimbabwe, Internal Medicine Unit, Faculty of Medicine and Health Sciences<br>Mugurungi, Owen; Ministry of Health and Child Care, AIDS and TB Unit<br>Machiha, Anna; Ministry of Health and Child Care, AIDS and TB Unit<br>Marks, Michael; London School of Hygiene & Tropical Medicine, Clinical Research Department; Hospital for Tropical Diseases,<br>Kranzer, Katharina; London School of Hygiene & Tropical Medicine, Clinical Research Department; Biomedical Research and Training Institute<br>Ferrand, Rashida; London School of Hygiene & Tropical Medicine, Department of Clinical Research; Biomedical Research and Training Institute
**Primary Subject Heading**:	Sexual health
Secondary Subject Heading:	Epidemiology, Global health, HIV/AIDS, Obstetrics and gynaecology, Public health
Keywords:	GENITOURINARY MEDICINE, HEALTH ECONOMICS, HIV & AIDS < INFECTIOUS DISEASES, Public health < INFECTIOUS DISEASES,

	MICROBIOLOGY, OBSTETRICS

SCHOLARONE™
Manuscripts

Investigating point-of-care diagnostics for sexually transmitted infections and antimicrobial resistance in antenatal care in Zimbabwe (IPSAZ): protocol for a mixed methods study

Kevin Martin[1 2 3], Chido Dziva Chikwari[2 4], Ethel Dauya[2], Constance Mackworth-Young[2 5], David Bath[5], Joseph D. Tucker[1], Victoria Simms[2 4], Tsitsi Bandason[2], Francis Ndowa,[6] Leolin Katsidzira[7], Owen Mugurungi[8], Anna Machiha[8], Michael Marks[1 9 10], Katharina Kranzer[1 2 11], Rashida A. Ferrand[1 2]

1. Department of Clinical Research, London School of Hygiene & Tropical Medicine, London, UK
2. Biomedical Research and Training Institute, Harare, Zimbabwe
3. Department of Global Health and Infection, Brighton and Sussex Medical School, Brighton, UK
4. Department of Infectious Disease Epidemiology, London School of Hygiene & Tropical Medicine, London, UK
5. Department of Global Health and Development, London School of Hygiene & Tropical Medicine, London, UK
6. Skin & Genito-Urinary Medicine Clinic, Harare, Zimbabwe
7. Internal Medicine Unit, Faculty of Medicine and Health Sciences, University of Zimbabwe, Harare, Zimbabwe
8. AIDS and TB unit, Ministry of Health and Child Care, Harare, Zimbabwe
9. Hospital for Tropical Diseases, University College London Hospital, London, United Kingdom
10. Division of Infection and Immunity, University College London, London, United Kingdom
11. Division of Infectious and Tropical Medicine, Medical Centre of the University of Munich, Munich, Germany

Corresponding author
Dr Kevin Martin
Department of Clinical Research
London School of Hygiene & Tropical Medicine
London WC1E 7HT, United Kingdom
kevin.martin@lshtm.ac.uk

Word counts: Manuscript = 4028. Abstract = 299

For peer review only - http://bmjopen.bmj.com/site/about/guidelines.xhtml

Abstract

Introduction

Sexually transmitted infections (STIs) can cause serious morbidity, including pelvic inflammatory disease, and adverse pregnancy outcomes. In low- and middle-income countries, limited laboratory infrastructure has resulted in a syndrome-based approach being used for management of STIs, which has poor sensitivity and specificity, leading to considerable underdiagnosis and overtreatment. The World Health Organization (WHO) has called for development and evaluation of strategies to inform replacement of syndromic management by diagnostic testing.

The aim of this project is to evaluate a strategy of point-of-care testing for six STIs in antenatal care (ANC) in Zimbabwe.

Methods and analysis

A prospective interventional study will be conducted in ANC clinics in Harare Province, Zimbabwe. One thousand pregnant women will be recruited when registering for routine antenatal care. Alongside routine HIV and syphilis testing, participants will be offered an integrated screening package including testing for *Chlamydia trachomatis* (CT), *Neisseria gonorrhoeae* (NG), *Trichomonas vaginalis* (TV), and Hepatitis B. All individuals with STIs will receive treatment, partner notification services, risk reduction counselling, and referral if needed according to national guidelines. Gonorrhoea samples will be cultured and tested for antimicrobial resistance as per WHO enhanced gonococcal antimicrobial surveillance programme guidelines.

The primary outcome measure is the composite prevalence of CT, NG, TV, syphilis and hepatitis B. A mixed methods process evaluation and economic evaluation will be conducted to understand the acceptability, feasibility, and cost-effectiveness of integrated STI testing, compared to standard of care (syndromic management).

Ethics and Dissemination

The study protocol was approved by the Medical Research Council of Zimbabwe, the Biomedical Research and Training Institute Institutional Review Board, and the London School of Hygiene & Tropical Medicine Research Ethics Committee. Results will be submitted to open-access peer-reviewed journals, presented at academic meetings and shared with participating communities and with national and international policy-making bodies.

For peer review only

For peer review only - http://bmjopen.bmj.com/site/about/guidelines.xhtml

Registration

https://clinicaltrials.gov/ct2/show/NCT05541081

Key words

Sexually transmitted infections; point-of-care testing; antenatal care; pregnancy; Zimbabwe

Article summary

Strengths and limitations of this study

- The mixed methods approach, with qualitative and quantitative data, will allow for the development of a multi-layered understanding of the acceptability and feasibility of this integrated screening package.
- Inclusion of an economic evaluation will allow for estimation of the cost-effectiveness of this screening package compared with routine care, which is essential for considering the scalability and sustainability of the programme.
- The large sample size will ensure an estimation of STI prevalence with high precision amongst antenatal attendees in Harare.
- This study does not include a formal outcome evaluation to assess the impact of this integrated screening package for STIs on adverse birth outcomes, which will need to be informed by future studies.
- Our focus on urban clinics will likely limit the generalisability of our findings to other urban centres in Southern Africa.

For peer review only

Introduction

Globally, there were an estimated 374 million infections of *Chlamydia trachomatis* (CT), *Neisseria gonorrhoeae* (NG), *Trichomonas vaginalis* (TV) and syphilis in 2020 among people aged 15 to 49 years.[1] Untreated, sexually transmitted infections (STIs) can cause adverse pregnancy outcomes, congenital infection and pelvic inflammatory disease.[2] STIs are also associated with increased risk of both HIV transmission and acquisiton.[3]

There are multiple contributing factors to the persistence of the high incidence of STIs globally. Complex sociocultural barriers such as stigma, limited sexual health education and barriers to condom use, and biomedical factors such as asymptomatic infections and increasing levels of antimicrobial resistance (AMR), have been exacerbated in recent years by a substandard global response to STIs, characterised by a lack of funding and political commitment.[1]

Effective management of STIs in low- and middle-income countries (LMIC) is additionally hindered by the use of syndromic management, which is the provision of treatment to an individual presenting with symptoms and/or signs that may be caused by an STI.[4] This is problematic as the majority of curable STIs are asymptomatic, particularly in women, and are missed by syndromic management.[5] Furthermore, treatment for infections that patients may not have may lead to side effects and increased AMR.[6]

Diagnostic platforms that do not require complicated laboratory infrastructure are available, but cost and lack of evidence on how they should be implemented to maximise both clinical effectiveness and cost-effectiveness in LMICs limit their implementation. Integration of diagnostic STI testing into health systems is likely to be key to reducing rates of STIs in LMICs and the World Health Organization (WHO) has called for evidence to inform replacement of syndromic management by diagnostic testing.[7, 8]

A group that may particularly benefit from the introduction of diagnostic testing for STIs is pregnant women. Pregnant women in LMICs are a high-risk population for STIs[9], and diagnosis and treatment may prevent adverse pregnancy outcomes and congenital transmission of some infections.[10] As point-of-care (POC) testing for HIV and syphilis has already been integrated into ANC services, this provides a platform for further STI testing and management and potentially enhances operational feasibility. In addition to curable STIs, this is also pertinent for hepatitis B, as testing is noted to be essential for the WHO's triple elimination initiative, which aims to eliminate

For peer review only - http://bmjopen.bmj.com/site/about/guidelines.xhtml

vertical transmission of HIV, syphilis, and hepatitis B.[11] Key to triple elimination is the provision of a multi-disciplinary approach within routine ANC services.

There is limited data on STI prevalence amongst pregnant women in Zimbabwe. Prevalence of CT up to 26.0%, of NG up to 6.4%, and TV up to 24.8% have been reported in pregnant women in South Africa and Zambia.[12, 13, 14, 15, 16] A 2010 study found a TV prevalence of 11.8% and syphilis prevalence of 1.2% among pregnant women in Harare.[17] Recent studies amongst female youth in Harare have demonstrated a combined CT/NG prevalence between 18.2% and 19.5%. [18, 19] A prevalence of hepatitis B ranging between 3.1-5.3% has been reported in pregnant women in South Africa [20, 21, 22].

Control of STIs, particularly NG, is additionally compromised by AMR.[23] Surveillance is key to identifying and monitoring AMR. WHO and the Centers for Disease Control, USA established the Enhanced Global Gonococcal Antimicrobial Surveillance Programme (EGASP) in 2015.[24] A sample of at least 100 gonococcal isolates per year per country is recommended.[23] However, gonococcal AMR data is still extremely limited and in 2018, only five of forty-seven countries in the WHO African Region reported susceptibility testing for NG of at least one of ceftriaxone, cefixime, ciprofloxacin and azithromycin.[25, 26, 27, 28]

Rationale

Evidence is required to inform the use of diagnostic testing for STIs in LMICs, at both national and international levels, particularly regarding acceptability, feasibility, and cost-effectiveness. Additionally, there is a data gap regarding the prevalence of STIs amongst pregnant women in Zimbabwe. Given the paucity of data on AMR in NG in Africa, there is also a need to strengthen AMR surveillance systems.

Aims and objectives

The overall aim of this study is to implement and evaluate a strategy for integration of POC diagnostics for STIs into ANC settings and to establish a gonococcal antimicrobial resistance surveillance strategy aligned with EGASP in Zimbabwe.

The objectives are to:

1. Determine the prevalence and yield of point-of-care testing for CT, NG, TV, syphilis and hepatitis B, and factors associated with presence of STIs, among pregnant women

For peer review only - http://bmjopen.bmj.com/site/about/guidelines.xhtml

For peer review only

2. Conduct a mixed-methods process evaluation to understand the acceptability and feasibility of POC STI testing and comprehensive case management in ANC settings

3. Estimate the cost and cost-effectiveness of integrated STI testing compared to standard of care

4. Investigate the prevalence of AMR for NG to inform the development of an EGASP in Zimbabwe

Methods and analysis

Study design and setting

A prospective interventional study will be conducted in primary healthcare clinics (PHCs) in Harare province, Zimbabwe. The PHCs are all based in urban, high-density settings, and provide nurse-led services including antenatal care and uncomplicated deliveries. High-risk women receive their ANC at central hospitals, with referral also available if complications develop in labour. Opt-out HIV and syphilis testing using rapid diagnostic tests is part of routine care. GeneXpert devices are often available for tuberculosis diagnosis, but the study will provide an additional machine to ensure that sufficient diagnostic capacity is available. We previously demonstrated the feasibility of using non-laboratory technicians to operate the GeneXpert device for on-site CT/NG testing in community settings in Bulawayo, Zimbabwe.[29]

Study population and recruitment

Pregnant women will be recruited when registering for routine antenatal care, starting in January 2023. It is the intention that only pregnant women attending their first antenatal care visit of this pregnancy will be recruited. However, if there is ongoing slower than expected recruitment, pregnant women attending for antenatal care follow-up visits will also be considered for enrolment. There will be no age cut-off for enrolment. Exclusion criteria will be enrolment in this study on a previous antenatal visit and being unable or unwilling to provide written informed consent. Recruitment will be conducted during weekdays only. Pregnant women will be consecutively enrolled as testing capacity allows.

Reasons for declining participation, and for exclusion, will be documented. If participants only consent to some of the STI tests, reasons for declining the others will be recorded.

For peer review only - http://bmjopen.bmj.com/site/about/guidelines.xhtml

Study procedures

The full schedule of events for pregnant women is described in Table 1. Following consent, an interviewer-administered questionnaire will collect sociodemographic data, clinical history including STI symptoms and recent antibiotic use, sexual and obstetric history. Contact information will be collected for follow-up.

For peer review only

For peer review only - http://bmjopen.bmj.com/site/about/guidelines.xhtml

Table 1: Schedule of events for pregnant women

Participants	Activity	Day 0 **(Study entry)**	Day 1-5	Day 4-14	On partner attendance	Birth	Post-partum **(planned telephone follow-up)**
All	Informed consent	X					
All	Questionnaire	X					
All	HIV testing	X					
All	Syphilis testing	X					
All	HBV testing	X					
All	Vaginal swab collection	X					
All	CT/NG testing	X					
All	TV testing	X					
STI test +ve	Health education	X					
HBV test +ve	Venepuncture	X					
HBV test +ve	HBV viral load and ALT testing	X					
HBV test +ve	Referral to secondary care	X					
HIV test +ve	Referral as per PHC processes	X					
CT/NG/TV/syphilis test +ve	Provision of treatment	X					
CT/NG/TV/syphilis test +ve	Partner notification advice and slip	X					

For peer review only - http://bmjopen.bmj.com/site/about/guidelines.xhtml

NG test +ve	Cervical swab collection	X					
NG test +ve	Plating of cervical swab & incubation at laboratory	X					
STI test +ve<br>Not treated on day 0	Contact participant by telephone and ask to return to PHC for treatment		X				
Cultured NG isolate	Storage of isolate at -80°C			X			
Partners	Provision of treatment to partners that attend PHC				X		
HBV test +ve	Provision of HBV birth dose vaccine co-ordinated with PHC and secondary care					X	
CT/NG/TV/syphilis test +ve	Contact by telephone to collect data on partner notification process						X
All	Contact by telephone to collect birth outcome data						X
All	Review of birth registry records to supplement birth outcome data from participants						X

ALT = Alanine aminotransferase. CT = Chlamydia. HBV = Hepatitis B virus. HIV = Human immunodeficiency virus. NG = Gonorrhoea. PHC = Primary healthcare centre. RDT = Rapid diagnostic test. STI = Sexually transmitted infection. TV = Trichomoniasis.

For peer review only

For peer review only - http://bmjopen.bmj.com/site/about/guidelines.xhtml

Participants will provide three self- or provider-taken vaginal swab samples. One vaginal swab sample will be tested for CT and NG using the Xpert® CT/NG assay (Cepheid), which has an analytic time of 90 minutes. The GeneXpert device will be operated using a rechargeable powerpack to provide an uninterrupted power supply. The second swab will be tested for TV using the OSOM® Trichomonas Rapid Test (Sekisui Diagnostics), which has an analytic time of 10 minutes.[30] The third swab will be stored for future studies including possible whole genome sequencing. A fingerprick blood sample will be taken for HIV, syphilis and hepatitis B testing using the SD BIOLINE HIV/Syphilis Duo (Abbott Diagnostics Medical Co. Ltd) (analytic time 20 minutes) and HBsAg 2 (Abbott Diagnostics Medical Co. Ltd) (analytic time 30 minutes) rapid tests, respectively.[31] HIV and syphilis testing, referral to HIV services, syphilis treatment, and partner notification for those with HIV or syphilis, are already part of routine care. The study team will work with health facility staff to integrate the additional STI testing with routine ANC services to prevent duplication of procedures.

Participants with positive test results and their partners will be managed in line with Zimbabwe national treatment guidelines.[32] For participants with an STI syndrome on presentation, immediate treatment will be provided for syndromes such as pelvic inflammatory disease and genital ulcer disease, where testing will not alter management. For vaginal discharge syndrome, participants will ideally wait for their results to receive tailored treatment, however they will receive metronidazole regardless of results, in order to cover for bacterial vaginosis. For symptomatic participants not willing or able to wait or return for their results, they will have the option to receive full syndromic treatment.

Participants will ideally collect their results and receive treatment if necessary within the same clinical visit. Participants who test positive for an STI but are unable to receive same day treatment, will be actively followed up by telephone, up to five times over a 28 day period, to advise them to return for treatment.

A client-referral approach will be used for notification of sexual partners. Women will be counselled on the importance of their partners receiving treatment and given partner notification (PN) slips for their partners to return for presumptive treatment. Although partners will be able to attend any clinic, treatment will be provided free-of-charge if they return to the study clinic.

Women newly diagnosed with HIV will be referred for antiretroviral therapy as per local PHC processes. Women newly diagnosed with hepatitis B will have hepatitis B viral load and alanine transaminase (ALT) testing, alongside referral to a gastroenterology specialist in secondary care. Although hepatitis B vaccination is currently included in the Zimbabwe national vaccination schedule

For peer review only - http://bmjopen.bmj.com/site/about/guidelines.xhtml

at six, ten, and fourteen weeks, birth dose vaccination is not yet standard of care in Zimbabwe. Birth dose vaccines will therefore be provided by the IPSAZ study. This is likely to be logistically complex and bespoke strategies for implementation will be designed in conjunction with healthcare teams at the individual PHCs and with local secondary care providers.

Birth outcome data will be collected from birth registers, including birthweight, gestation, mode of delivery, and still birth. Estimated due date, which will be compared with actual birth date to determine prematurity, will be based on last menstrual period. Participants will also be contacted by telephone post-partum to facilitate this process by providing information on date and location of birth, and to provide supplemental data if necessary if the birth register is incomplete. Participants will also be asked about number of ANC visits, and for women with positive STI results, if they gave the PN slip to their partner, and if their partners were treated.

Process evaluation

A mixed-methods process evaluation will be conducted, based on the MRC Process Evaluation Framework.[33] The focus will be on understanding what was implemented and how; how the intervention led to change; and how local context affects implementation and shapes outcomes. Steckler and Linnan's process evaluation framework has also guided the choice of specific research domains related to implementation, where fidelity, dose and reach/coverage, are central features.[34]

Table 2 details the process evaluation research domains and questions. A logic model demonstrating the proposed theory of change is shown in Figure 1.

For peer review only - http://bmjopen.bmj.com/site/about/guidelines.xhtml

For peer review only - http://bmjopen.bmj.com/site/about/guidelines.xhtml

Table 2: Framework for process evaluation.

**Framework domain**	**Research domain**	**Research questions**	**Data collection methods and sources**
Implementation	Fidelity	• How did implementation vary from the protocol i.e. (a) offering STI screening, (b) undertaking STI screening, (c) providing comprehensive case management including partner notification, (d) training and supervision of staff<br>• What were the barriers and facilitators to implementation?<br>• What adaptations were made?	Routine monitoring data<br>Structured observation<br>FGDs with clinic staff (1-2 per PHC) and IDIs with research team (all members), pregnant women (8-10 per PHC), partners (3-4 per PHC) and clinic staff (4 per PHC) *<br>IDIs with pregnant women who decline STI screening (3-4 per PHC) to explore reasons for this.
	Coverage	• What proportion of: (a) Pregnant women attending antenatal care were offered STI screening, (b) Pregnant women who were offered STI screening took it up (c) Positive STI cases were treated, (d) Partners of positive cases were treated?<br>• How equitable was this coverage?<br>• What were the barriers and facilitators to each step?	
Mechanisms of Impact	Responses to and interactions with the intervention	• Which components of the intervention were best accepted and adopted by pregnant women and HCWs and why?<br>• What challenges and barriers were faced?	Structured Observation<br>FGDs and IDIs with pregnant women, partners, and HCWs*
	Interactions and Consequences	• How did various components of the intervention interact?<br>• Were there any unanticipated pathways or consequences?	

For peer review only - http://bmjopen.bmj.com/site/about/guidelines.xhtml

Context	Proximal and distal	• What social, cultural, political, and logistical factors impede or facilitate how the intervention was implemented, and how were HCWs able to engage with and adopt aspects of the intervention?<br>• What were contextual reasons for adaptations to the intervention and its delivery?	FGDs and IDIs with pregnant women, partners, and HCWs*<br>Structured and unstructured observations<br>Key informant interviews with local health authorities, and community leaders<br>Context diaries to record external events

STI = Sexually transmitted infection. HCW = Healthcare worker. IDI = In-depth interview. FGD = Focus group discussion. PHC = Primary healthcare clinic.

*Number of IDIs/FGDs listed are approximations.

For peer review only

Following initial qualitative formative work to refine the testing strategy, a concurrent triangulation strategy will be used, with quantitative and qualitative data collected in parallel, with similar weighting given to each.[35] This will allow for triangulation of data in order to comprehensively address the process evaluation questions. Routine monitoring data will include uptake of testing, treatment, and partner notification, as well as stock monitoring, debriefing minutes, and recording of GeneXpert error codes. Data collection will also include structured and unstructured observation, and focus group discussions and in-depth interviews with key stakeholders including pregnant women, partners, clinic staff, policymakers, and the research team involved in delivery of the intervention (including both the clinic-based team and laboratory staff). Different topic guides will be designed for interviews and focus group discussions at different stages of implementation, to reflect the changing focus of the process evaluation. Participants for interviews and group discussions will be purposively selected to ensure a relevant range of views for each stage of the process evaluation. Adaptations will be made to the testing strategy based on interim process evaluation findings to improve ongoing implementation.

Thematic analysis on qualitative data will be performed to identify and develop key themes and concepts on addressing what was implemented and how; how the intervention led to change; and how local context affects implementation and shapes outcomes.

Economic evaluation

Cost-effectiveness will be evaluated as the ratio of incremental costs and incremental effects of point-of-care testing for CT, NG, and TV, in comparison with routine care using syndromic management. Total costs and effects will be estimated for each arm, with incremental values calculated from these estimates.

Clinical endpoints include mild infection, pelvic inflammatory disease, infertility, and adverse birth outcomes including miscarriage, stillbirth, low birthweight, and prematurity. Incidence of clinical endpoints will be estimated from existing literature, with such estimates for adverse birth outcomes being supported by primary data collection. Disability-adjusted life years will be the main outcome, and will be modelled based on clinical endpoints, predicted life expectancy and disability weights.[36] Intermediate measures include cost per patient screened, diagnosed with an STI, and treated. A simple static decision tree model will be used to structure the cost-effectiveness evaluation, which will be from a health system perspective.

Health system costs required to deliver the testing strategy will be collected from both primary and secondary data sources using a bespoke cost extraction tool. Financial and economic costs will be

For peer review only - http://bmjopen.bmj.com/site/about/guidelines.xhtml

estimated based on study financial records, staff interviews, time-and-motion studies, and other sources such as national salary scales for the Zimbabwe health service. Costs categories will include start-up costs such as training costs, capital costs including buildings and storage and equipment, and recurrent costs, including personnel, consumables, and transport costs. Costs associated with downstream complications of infection will be estimated from the literature. Costs incurred by patients whilst attending ANC, including transport costs and opportunity costs, will be collected using the interviewer-administered questionnaire. Additional costs associated with the testing strategy, such as a longer visit or to return for treatment, will be recorded.

Individual and combined parameter uncertainty will be investigated using deterministic and probabilistic sensitivity analyses. The cost-effectiveness of the testing strategy compared to syndromic management will be compared against appropriate cost-effectiveness thresholds for Zimbabwe.

Antimicrobial resistance surveillance

EGASP recommends the collection of at least 100 gonococcal isolates per year per country. Assuming a prevalence of NG of 3% amongst pregnant women, only 30 women with NG will be identified. To supplement the number of isolates obtained from ANC participants, men aged 18 years and above presenting with urethral discharge to the study clinics who provide written informed consent will be recruited. Consecutive sampling will be performed as resources allow, with the antenatal testing strategy taking priority.

Men with urethral discharge have been selected as the yield of a positive gonococcal culture will be high. Assuming an estimate of 50% NG prevalence amongst symptomatic men,[37] we anticipate recruiting approximately 140 males with urethral discharge. We therefore estimate isolation of 70 gonococcal isolates from men which, in addition to 30 isolates from women, will give 100 NG isolates in total. If the prevalence of NG is lower than predicted, recruitment of men with urethral discharge will continue until 100 NG isolates have been obtained.

Data on age, sex, risk factors for STIs including number of recent sexual partners and gender of sexual partners, current STI symptoms, recent antibiotic use, treatment provided at the PHC, and previous STI diagnoses including HIV, will be collected using an interview-administered questionnaire. Nurse-collected urethral swabs will be collected from all enrolled men, and they will be managed syndromically according to national guidelines as part of routine care.

For peer review only - http://bmjopen.bmj.com/site/about/guidelines.xhtml

Testing procedures for antimicrobial resistance

WHO EGASP guidelines and standard operating procedures will be adapted for use with pregnant women, and otherwise followed for AMR testing for NG.[38] Nurse-collected cervical samples will be obtained using ESwabs® (COPAN Diagnostics Inc.) from women initially found to be positive for NG using the Xpert assay. Speculum examinations will be conducted by nurses with many years of experience performing these examinations, using plastic disposable speculums. Samples will be plated onto the InTray® GC (Biomed Diagnostics Inc.) in-vitro device, as will urethral samples collected from men. These will be subsequently incubated in the laboratory and minimum inhibitory concentrations of ceftriaxone, cefixime, azithromycin and ciprofloxacin will be determined using Etest® (bioMérieux) and interpreted using the European Committee on Antimicrobial Susceptibility Testing (EUCAST) standards.[39]

Sample size calculations

There is limited data on STI prevalence in pregnant women in Zimbabwe. A prevalence of curable STIs between 32.0% and 37.0% has been reported in South Africa and Zambia.[12, 13, 14, 15, 16] Our recent studies among female youth in Harare have demonstrated combined CT/NG prevalence between 18.2% and 19.5%. [18, 19] The prevalence of hepatitis B in pregnant women in South Africa has ranged between 3.1% and 5.3%.[20, 21, 22] Therefore, a conservative estimate of composite prevalence of curable STIs and hepatitis B is 30.0%. With a desired precision of 3% and alpha of 0.05, a sample size of 896 is required. To allow for invalid test results, 1000 pregnant women will be screened. The minimum detectable odds ratios for factors associated with the presence of STIs at different composite prevalence of STIs are shown in Table 3.

Table 3: Minimum detectable odds ratios for factors associated with presence of STIs

Sample size*	STI prevalence	Precision	Smallest OR detected at 80% power	Smallest OR detected at 90% power
896	20%	2.6%	1.74	1.89
	25%	2.8%	1.67	1.80
	30%	3.0%	1.63	1.75

*Assuming a prevalence of a risk factor of 20% amongst those without STIs. OR = Odds ratio. STI = Sexually transmitted infection.

For peer review only - http://bmjopen.bmj.com/site/about/guidelines.xhtml

Statistical analysis

The primary outcome measure is the composite prevalence of CT, NG, TV, syphilis and hepatitis B in this population.

Other outcome measures include:
1) Individual prevalence of each STI (CT, NG, TV, syphilis, hepatitis B, and HIV)
2) Uptake of testing
3) Uptake of treatment
4) Uptake of partner notification
5) STI yield (number of participants with a positive STI result/total number of eligible individuals)
6) Prevalence of antimicrobial resistance to ceftriaxone, cefixime, azithromycin and ciprofloxacin in NG isolates
7) Prevalence of preterm birth, miscarriage, and low birth weight

Categorical variables will be described using frequencies and percentages. Continuous variables will be described using either mean (standard deviation) or median (interquartile range) for normally distributed and non-normally distributed data, respectively. Multivariable logistic regression will be used to assess factors associated with presence of STIs. Clustering will be adjusted for at clinic level. Logistic regression will also be used to assess the relationship between STI diagnosis and birth outcomes.

Data management procedures

Quantitative data will be collected using electronic case report forms on tablet computers using Open Data Kit software, with range restrictions and dropdown menus too minimise data entry errors. CT/NG results recorded on tablet computers will be cross-checked with the readout from the GeneXpert device. Data will be managed and cleaned using STATA (StataCorp, Texas, USA). Interviews and focus group discussions will be audio recorded, and transcribed verbatim. Anonymisation of transcripts will be performed once translation and transcription has been completed.

All data will be stored in password-controlled databases, and all data will be encrypted. All data collected will be anonymised using a unique study ID. Any identifiable data (e.g., locator forms and consent forms) will be stored in secure, locked facilities with access limited to the study team.

For peer review only - http://bmjopen.bmj.com/site/about/guidelines.xhtml

Data sharing

The informed consent procedure will clarify the sharing of anonymised data, either via a public data repository, or by directly sharing with other researchers.

At the time of publication of research, the subset of data required for the purposes of verifying research findings will be made available for sharing and will be placed in Data Compass (the LSHTM institutional research data repository – accessible at https://datacompass.lshtm.ac.uk/). This repository will enable direct download of records with codebooks to enable replication of the data analyses. A more complete sharing of data with any research group requesting access to individual data records will be done 12 months after publication. At this point, all data and study tools will be made available through Data Compass. Data for sharing will be de-identified prior to release. Details of how to access data will be published with each study publication.

Patient and public involvement

Initial formative work will include dialogue and input from service users to help refine the testing strategy and data collection tools. Findings will also be disseminated through study clinics.

Ethics and dissemination

The study protocol has been approved by the Medical Research Council of Zimbabwe (MRCZ/A/2899), the Biomedical Research and Training Institute Institutional Review Board (AP176/2022) and the London School of Hygiene & Tropical Medicine Research Ethics Committee (26787). The completed SPIRIT (Standard Protocol Items: Recommendations for Interventional Trials) checklist can be found in the supplemental material (supplementary file A).

Written informed consent to participate in the study will be obtained in the preferred language of the potential participant (English or Shona). Specific consent will also be sought for storage of samples and the sharing of anonymised data via a public data repository. Participants will not be identifiable from this information. An example participant informed consent form for enrolment into the main STI testing study can be found in the supplemental material (supplementary file B).

In Zimbabwe, individuals who are under 18 years of age and pregnant, are considered emancipated minors. Therefore, independent informed consent will be obtained from pregnant minors.

For peer review only - http://bmjopen.bmj.com/site/about/guidelines.xhtml

For any participants who are under 16 years of age and pregnant, we will consider on an individual basis whether further input is needed regarding child protection. This may include discussion of the case with a multidisciplinary team and possible referral to social services. If these instances do arise, we aim to integrate into existing clinic processes as much as possible.

Results will be submitted to open-access peer-reviewed journals, presented at academic meetings and shared with participating communities and with national and international policy-making bodies.

Discussion

There is mounting evidence that syndromic management is not an effective method for the control of STIs.[14, 40, 41] However, evidence is required by national and international policymakers to inform how to transition towards diagnostic testing for STIs. We anticipate high prevalence of STIs among pregnant women in this study, thus providing additional evidence that new strategies are required for control of STIs in Zimbabwe. The data on acceptability, feasibility, and cost-effectiveness, will provide guidance on how best to implement new strategies for a package of integrated diagnostic testing in antenatal care. Furthermore, this study will provide baseline data for the design of future testing strategies, implemented at a larger scale, and potentially as part of a cluster-randomised trial to demonstrate efficacy.

The IPSAZ study has several strengths. The large sample size ensures that STI prevalence in the antenatal population in Harare will be estimated with high precision. The use of mixed methods for the process evaluation will enable quantitative and qualitative data to complement and inform each other. Furthermore, the economic evaluation will inform policymakers about whether introducing such a testing programme represents a cost-effective use of health resources in this and similar contexts. Finally, we will be following adapted EGASP standard procedures for gonococcal culture and AMR testing. EGASP guidelines do not currently include provision for sample collection from pregnant women, and are focussed on symptomatic men. Our experience will therefore inform the potential expansion of EGASP guidelines to include pregnant women, and to consider the use of antenatal networks as a platform for gonococcal surveillance for future national EGASP programmes.

We acknowledge some limitations. There is no formal outcome evaluation comparing birth outcomes between those who received the intervention, and those who did not. This is an important research question. Although treatment of STIs is likely to prevent complications such as pelvic inflammatory disease, there is conflicting evidence on whether provision of treatment for STIs during pregnancy

For peer review only - http://bmjopen.bmj.com/site/about/guidelines.xhtml

prevents adverse birth outcomes.[42, 43, 44] Future studies need to investigate the clinical effectiveness of scaling-up of STI testing and treatment in antenatal care.

The testing strategy will be delivered by members of a dedicated study team, which may not be representative of how testing would integrate into existing governmental clinical services, especially given that resources and staff are severely constrained in Zimbabwe. However, the mixed methods approach to the process evaluation will provide supporting information to contextualise the findings.

Finally, the study will be conducted in urban PHCs in Harare province. As a result, the results will likely be generalisable to urban centres in Southern Africa, but less so to rural areas in this region.

In summary, the IPSAZ study will provide important data to develop an integrated screening package for STIs in antenatal care in Southern Africa. The epidemiological data, process evaluation, and economic evaluation will all help inform sustainability and scalability, in order to provide evidence-based policy recommendations. Additionally, the collection of gonococcal AMR data will also be used to inform national STI treatment guidelines in Zimbabwe.

Data availability statement

No data is associated with this paper. Following publication of study results, the datasets that will be used and/or analysed during this study will be available from the corresponding author on reasonable request. Additionally, individual, anonymised participant data and a data dictionary will be available through The London School of Hygiene & Tropical Medicine repository (Data Compass) 12 months after publication of results.

Author contributions

KM conceptualised the IPSAZ study, supervised by RAF, KK, and MM. KM developed the first draft of the paper, developed the study manual of operations, and will be responsible for study implementation. KM and TB are responsible for data management. CDC, ED, CMY, DB, JT, VS, TB, FN, LK, OM, and AM, provided guidance on study design and methodology. All authors reviewed the final draft of the manuscript.

Funding statement

This work was supported by the Wellcome Trust grant number 225468/Z/22/Z awarded to KM. The funder of the study has had no role in study design, and will have no role in data collection, data analysis, data interpretation, writing of the report, or decision to submit.

For peer review only - http://bmjopen.bmj.com/site/about/guidelines.xhtml

Competing interests

No competing interests were disclosed.

For peer review only

For peer review only - http://bmjopen.bmj.com/site/about/guidelines.xhtml

References

1. World Health Organization. Global health sector strategies on, respectively, HIV, viral hepatitis and sexually transmitted infections for the period 2022-2030. 2022.
2. Rowley J, Vander Hoorn S, Korenromp E, Low N, Unemo M, Abu-Raddad LJ, et al. Chlamydia, gonorrhoea, trichomoniasis and syphilis: global prevalence and incidence estimates, 2016. Bull World Health Organ. 2019;97(8):548-62P.
3. Fleming DT, Wasserheit JN. From epidemiological synergy to public health policy and practice: the contribution of other sexually transmitted diseases to sexual transmission of HIV infection. Sex Transm Infect. 1999;75(1):3-17.
4. World Health Organization. Guidelines for the management of symptomatic sexually transmitted infections. 2021.
5. Farley TA, Cohen DA, Elkins W. Asymptomatic sexually transmitted diseases: the case for screening. Prev Med. 2003;36(4):502-9.
6. Peeling RW, Holmes KK, Mabey D, Ronald A. Rapid tests for sexually transmitted infections (STIs): the way forward. Sex Transm Infect. 2006;82 Suppl 5(Suppl 5):v1-6.
7. Toskin I, Blondeel K, Peeling RW, Deal C, Kiarie J. Advancing point of care diagnostics for the control and prevention of STIs: the way forward. Sex Transm Infect. 2017;93(S4):S81-s8.
8. World Health Organization. Global Health Sector Strategy on sexually transmitted infections 2016-2021. 2016.
9. Joseph Davey DL, Shull HI, Billings JD, Wang D, Adachi K, Klausner JD. Prevalence of Curable Sexually Transmitted Infections in Pregnant Women in Low- and Middle-Income Countries From 2010 to 2015: A Systematic Review. Sex Transm Dis. 2016;43(7):450-8.
10. Mullick S, Watson-Jones D, Beksinska M, Mabey D. Sexually transmitted infections in pregnancy: prevalence, impact on pregnancy outcomes, and approach to treatment in developing countries. Sex Transm Infect. 2005;81(4):294-302.
11. Cohn J, Owiredu MN, Taylor MM, Easterbrook P, Lesi O, Francoise B, et al. Eliminating mother-to-child transmission of human immunodeficiency virus, syphilis and hepatitis B in sub-Saharan Africa. Bull World Health Organ. 2021;99(4):287-95.
12. Moodley D, Moodley P, Sebitloane M, Soowamber D, McNaughton-Reyes HL, Groves AK, et al. High prevalence and incidence of asymptomatic sexually transmitted infections during pregnancy and postdelivery in KwaZulu Natal, South Africa. Sex Transm Dis. 2015;42(1):43-7.
13. Green H, Taleghani S, Nyemba D, Myer L, Davey DJ. Partner notification and treatment for sexually transmitted infections among pregnant women in Cape Town, South Africa. Int J STD AIDS. 2020;31(13):1282-90.
14. Nyemba DC, Medina-Marino A, Peters RPH, Klausner JD, Ngwepe P, Myer L, et al. Prevalence, incidence and associated risk factors of STIs during pregnancy in South Africa. Sex Transm Infect. 2020.
15. Joseph Davey DL, Nyemba DC, Gomba Y, Bekker LG, Taleghani S, DiTullio DJ, et al. Prevalence and correlates of sexually transmitted infections in pregnancy in HIV-infected and-uninfected women in Cape Town, South Africa. PLoS One. 2019;14(7):e0218349.
16. Chaponda EB, Chico RM, Bruce J, Michelo C, Vwalika B, Mharakurwa S, et al. Malarial Infection and Curable Sexually Transmitted and Reproductive Tract Infections Among Pregnant Women in a Rural District of Zambia. Am J Trop Med Hyg. 2016;95(5):1069-76.
17. Kurewa NE, Mapingure MP, Munjoma MW, Chirenje MZ, Rusakaniko S, Stray-Pedersen B. The burden and risk factors of Sexually Transmitted Infections and Reproductive Tract Infections among pregnant women in Zimbabwe. BMC Infectious Diseases. 2010;10(1):127.
18. Martin K, Olaru ID, Buwu N, Bandason T, Marks M, Dauya E, et al. Uptake of and factors associated with testing for sexually transmitted infections in community-based settings among youth in Zimbabwe: a mixed-methods study. Lancet Child Adolesc Health. 2021.
19. Kranzer K, Simms V, Dauya E, Olaru ID, Dziva Chikwari C, Martin K, et al. Identifying youth at high risk for sexually transmitted infections in community-based settings using a risk prediction tool: a validation study. BMC Infectious Diseases. 2021;21(1):1234.

For peer review only - http://bmjopen.bmj.com/site/about/guidelines.xhtml

20. Chotun N, Preiser W, van Rensburg CJ, Fernandez P, Theron GB, Glebe D, et al. Point-of-care screening for hepatitis B virus infection in pregnant women at an antenatal clinic: A South African experience. PLoS One. 2017;12(7):e0181267.
21. Andersson MI, Maponga TG, Ijaz S, Barnes J, Theron GB, Meredith SA, et al. The epidemiology of hepatitis B virus infection in HIV-infected and HIV-uninfected pregnant women in the Western Cape, South Africa. Vaccine. 2013;31(47):5579-84.
22. Thumbiran NV, Moodley D, Parboosing R, Moodley P. Hepatitis B and HIV co-infection in pregnant women: indication for routine antenatal hepatitis B virus screening in a high HIV prevalence setting. S Afr Med J. 2014;104(4):307-9.
23. Wi T, Lahra MM, Ndowa F, Bala M, Dillon JR, Ramon-Pardo P, et al. Antimicrobial resistance in Neisseria gonorrhoeae: Global surveillance and a call for international collaborative action. PLoS Med. 2017;14(7):e1002344.
24. Weston EJ, Wi T, Papp J. Strengthening Global Surveillance for Antimicrobial Drug-Resistant Neisseria gonorrhoeae through the Enhanced Gonococcal Antimicrobial Surveillance Program. Emerg Infect Dis. 2017;23(13):S47-52.
25. Latif AS, Gwanzura L, Machiha A, Ndowa F, Tarupiwa A, Gudza-Mugabe M, et al. Antimicrobial susceptibility in Neisseria gonorrhoeae isolates from five sentinel surveillance sites in Zimbabwe, 2015-2016. Sex Transm Infect. 2018;94(1):62-6.
26. Unemo M, Lahra MM, Cole M, Galarza P, Ndowa F, Martin I, et al. World Health Organization Global Gonococcal Antimicrobial Surveillance Program (WHO GASP): review of new data and evidence to inform international collaborative actions and research efforts. Sex Health. 2019;16(5):412-25.
27. Takuva S, Mugurungi O, Mutsvangwa J, Machiha A, Mupambo AC, Maseko V, et al. Etiology and antimicrobial susceptibility of pathogens responsible for urethral discharge among men in Harare, Zimbabwe. Sex Transm Dis. 2014;41(12):713-7.
28. WHO Global Health Observatory. WHO Gonococcal AMR Surveillance Programme [Available from: https://www.who.int/data/gho/data/themes/topics/who-gonococcal-amr-surveillance-programme-who-gasp.
29. Martin K, Dziva Chikwari C, Mackworth-Young CRS, Chisenga M, Bandason T, Dauya E, et al. “It was difficult to offer same day results”: evaluation of community-based point-of-care testing for sexually transmitted infections among youth using the GeneXpert platform in Zimbabwe. BMC Health Services Research. 2022;22(1):171.
30. Gaydos CA, Klausner JD, Pai NP, Kelly H, Coltart C, Peeling RW. Rapid and point-of-care tests for the diagnosis of Trichomonas vaginalis in women and men. Sex Transm Infect. 2017;93(S4):S31-s5.
31. World Health Organization. Public reports of WHO prequalified IVDs [Available from: https://www.who.int/diagnostics_laboratory/evaluations/pq-list/hbsag/public_report/en/.
32. Zimbabwe Ministry of Health and Child Care. National STI Management Guidelines. 2019.
33. Moore GF, Audrey S, Barker M, Bond L, Bonell C, Hardeman W, et al. Process evaluation of complex interventions: Medical Research Council guidance. BMJ : British Medical Journal. 2015;350:h1258.
34. Linnan L, Steckler A. Process evaluation for public health interventions and research. San Francisco: Jossey-Bass; 2002.
35. Creswell JW. Research Design: Qualitative, Quantitative, and Mixed Methods Approaches: Sage; 2011.
36. GBD 2019 Diseases and Injuries Collaborators. Global burden of 369 diseases and injuries in 204 countries and territories, 1990-2019: a systematic analysis for the Global Burden of Disease Study 2019. Lancet. 2020;396(10258):1204-22.
37. Rietmeijer CA, Mungati M, Machiha A, Mugurungi O, Kupara V, Rodgers L, et al. The Etiology of Male Urethral Discharge in Zimbabwe: Results from the Zimbabwe STI Etiology Study. Sex Transm Dis. 2018;45(1):56-60.
38. World Health Organization. Enhanced Gonococcal Antimicrobial Surveillance Programme (EGASP): General Protocol. 2021.
39. The European Committee on Antimicrobial Susceptibility Testing. Breakpoint tables for interpretation of MICs and zone diameters. Version 8.0 2022 [Available from:

For peer review only - http://bmjopen.bmj.com/site/about/guidelines.xhtml

https://www.eucast.org/fileadmin/src/media/PDFs/EUCAST_files/Breakpoint_tables/v_12.0_Breakpoint_Tables.pdf.
40. Garrett NJ, Osman F, Maharaj B, Naicker N, Gibbs A, Norman E, et al. Beyond syndromic management: Opportunities for diagnosis-based treatment of sexually transmitted infections in low- and middle-income countries. PLoS ONE. 2018;13(4):e0196209.
41. Verwijs MC, Agaba SK, Sumanyi JC, Umulisa MM, Mwambarangwe L, Musengamana V, et al. Targeted point-of-care testing compared with syndromic management of urogenital infections in women (WISH): a cross-sectional screening and diagnostic accuracy study. Lancet Infect Dis. 2019;19(6):658-69.
42. Ravindran J, Richardson BA, Kinuthia J, Unger JA, Drake AL, Osborn L, et al. Chlamydia, Gonorrhea, and Incident HIV Infection During Pregnancy Predict Preterm Birth Despite Treatment. J Infect Dis. 2021;224(12):2085-93.
43. Folger AT. Maternal Chlamydia trachomatis infections and preterm birth:the impact of early detection and eradication during pregnancy. Matern Child Health J. 2014;18(8):1795-802.
44. Liu B, Roberts CL, Clarke M, Jorm L, Hunt J, Ward J. Chlamydia and gonorrhoea infections and the risk of adverse obstetric outcomes: a retrospective cohort study. Sex Transm Infect. 2013;89(8):672-8.

For peer review only

For peer review only - http://bmjopen.bmj.com/site/about/guidelines.xhtml

Figure 1: Logic model for mixed methods process evaluation

For peer review only

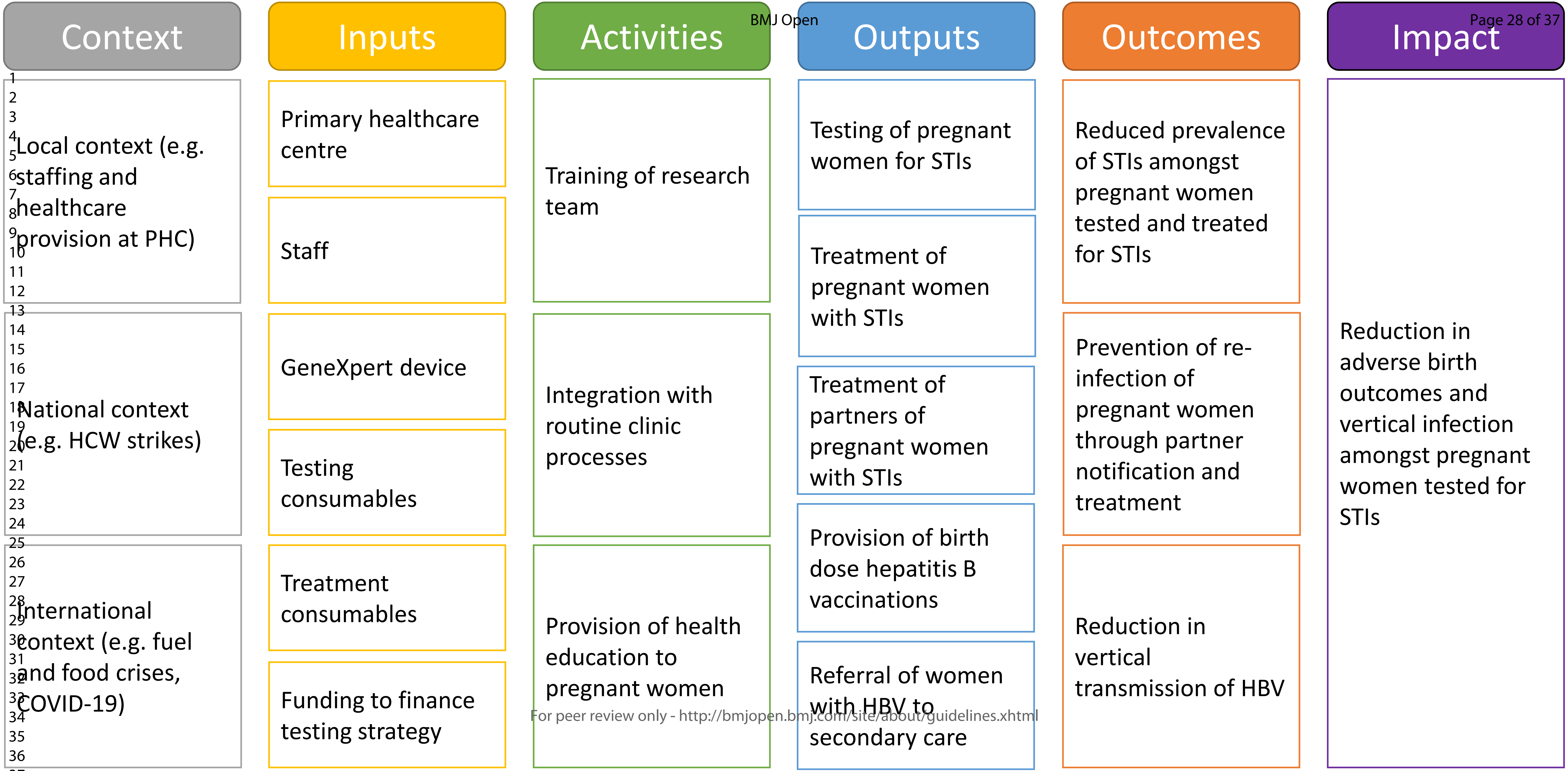
Context
Local context (e.g. staffing and healthcare provision at PHC)
National context (e.g. HCW strikes)
International context (e.g. fuel and food crises, COVID-19)
Inputs
Primary healthcare centre
Staff
GeneXpert device
Testing consumables
Treatment consumables
Funding to finance testing strategy
Activities
Training of research team
Integration with routine clinic processes
Provision of health education to pregnant women
Outputs
Testing of pregnant women for STIs
Treatment of pregnant women with STIs
Treatment of partners of pregnant women with STIs
Provision of birth dose hepatitis B vaccinations
Referral of women with HBV to secondary care
Outcomes
Reduced prevalence of STIs amongst pregnant women tested and treated for STIs
Prevention of re-infection of pregnant women through partner notification and treatment
Reduction in vertical transmission of HBV
Impact
Reduction in adverse birth outcomes and vertical infection amongst pregnant women tested for STIs

SPIRIT
STANDARD PROTOCOL ITEMS: RECOMMENDATIONS FOR INTERVENTIONAL TRIALS

SPIRIT 2013 Checklist: Recommended items to address in a clinical trial protocol and related documents*

Section/item	Item No	Description	Addressed on page number
**Administrative information**			
Title	1	Descriptive title identifying the study design, population, interventions, and, if applicable, trial acronym	______1______
Trial registration	2a	Trial identifier and registry name. If not yet registered, name of intended registry	______3______
	2b	All items from the World Health Organization Trial Registration Data Set	______N/A____
Protocol version	3	Date and version identifier	______N/A____
Funding	4	Sources and types of financial, material, and other support	______20_____
Roles and responsibilities	5a	Names, affiliations, and roles of protocol contributors	______20_____
	5b	Name and contact information for the trial sponsor	_______N/A___
	5c	Role of study sponsor and funders, if any, in study design; collection, management, analysis, and interpretation of data; writing of the report; and the decision to submit the report for publication, including whether they will have ultimate authority over any of these activities	_______20____
	5d	Composition, roles, and responsibilities of the coordinating centre, steering committee, endpoint adjudication committee, data management team, and other individuals or groups overseeing the trial, if applicable (see Item 21a for data monitoring committee)	______N/A_____

For peer review only - http://bmjopen.bmj.com/site/about/guidelines.xhtml

Introduction

Background and rationale	6a	Description of research question and justification for undertaking the trial, including summary of relevant studies (published and unpublished) examining benefits and harms for each intervention	______4-5____
	6b	Explanation for choice of comparators	______N/A____
Objectives	7	Specific objectives or hypotheses	______5-6_____
Trial design	8	Description of trial design including type of trial (eg, parallel group, crossover, factorial, single group), allocation ratio, and framework (eg, superiority, equivalence, noninferiority, exploratory)	______N/A_____

Methods: Participants, interventions, and outcomes

Study setting	9	Description of study settings (eg, community clinic, academic hospital) and list of countries where data will be collected. Reference to where list of study sites can be obtained	_______6______
Eligibility criteria	10	Inclusion and exclusion criteria for participants. If applicable, eligibility criteria for study centres and individuals who will perform the interventions (eg, surgeons, psychotherapists)	_______6______
Interventions	11a	Interventions for each group with sufficient detail to allow replication, including how and when they will be administered	_______7-11____
	11b	Criteria for discontinuing or modifying allocated interventions for a given trial participant (eg, drug dose change in response to harms, participant request, or improving/worsening disease)	_______N/A_____
	11c	Strategies to improve adherence to intervention protocols, and any procedures for monitoring adherence (eg, drug tablet return, laboratory tests)	_______N/A_____
	11d	Relevant concomitant care and interventions that are permitted or prohibited during the trial	_______7-11____
Outcomes	12	Primary, secondary, and other outcomes, including the specific measurement variable (eg, systolic blood pressure), analysis metric (eg, change from baseline, final value, time to event), method of aggregation (eg, median, proportion), and time point for each outcome. Explanation of the clinical relevance of chosen efficacy and harm outcomes is strongly recommended	______17_______
Participant timeline	13	Time schedule of enrolment, interventions (including any run-ins and washouts), assessments, and visits for participants. A schematic diagram is highly recommended (see Figure)	______8-9_____

For peer review only - http://bmjopen.bmj.com/site/about/guidelines.xhtml

Sample size	14	Estimated number of participants needed to achieve study objectives and how it was determined, including clinical and statistical assumptions supporting any sample size calculations	______16_____
Recruitment	15	Strategies for achieving adequate participant enrolment to reach target sample size	_______N/A____

Methods: Assignment of interventions (for controlled trials)

Allocation:

Sequence generation	16a	Method of generating the allocation sequence (eg, computer-generated random numbers), and list of any factors for stratification. To reduce predictability of a random sequence, details of any planned restriction (eg, blocking) should be provided in a separate document that is unavailable to those who enrol participants or assign interventions	_______N/A____
Allocation concealment mechanism	16b	Mechanism of implementing the allocation sequence (eg, central telephone; sequentially numbered, opaque, sealed envelopes), describing any steps to conceal the sequence until interventions are assigned	_______N/A____
Implementation	16c	Who will generate the allocation sequence, who will enrol participants, and who will assign participants to interventions	_______N/A____
Blinding (masking)	17a	Who will be blinded after assignment to interventions (eg, trial participants, care providers, outcome assessors, data analysts), and how	_______N/A____
	17b	If blinded, circumstances under which unblinding is permissible, and procedure for revealing a participant's allocated intervention during the trial	_______N/A____

Methods: Data collection, management, and analysis

Data collection methods	18a	Plans for assessment and collection of outcome, baseline, and other trial data, including any related processes to promote data quality (eg, duplicate measurements, training of assessors) and a description of study instruments (eg, questionnaires, laboratory tests) along with their reliability and validity, if known. Reference to where data collection forms can be found, if not in the protocol	_______17______
	18b	Plans to promote participant retention and complete follow-up, including list of any outcome data to be collected for participants who discontinue or deviate from intervention protocols	______N/A_____

For peer review only - http://bmjopen.bmj.com/site/about/guidelines.xhtml

Data management	19	Plans for data entry, coding, security, and storage, including any related processes to promote data quality (eg, double data entry; range checks for data values). Reference to where details of data management procedures can be found, if not in the protocol	______17______
Statistical methods	20a	Statistical methods for analysing primary and secondary outcomes. Reference to where other details of the statistical analysis plan can be found, if not in the protocol	______17_______
	20b	Methods for any additional analyses (eg, subgroup and adjusted analyses)	______17_______
	20c	Definition of analysis population relating to protocol non-adherence (eg, as randomised analysis), and any statistical methods to handle missing data (eg, multiple imputation)	______N/A______
**Methods: Monitoring**			
Data monitoring	21a	Composition of data monitoring committee (DMC); summary of its role and reporting structure; statement of whether it is independent from the sponsor and competing interests; and reference to where further details about its charter can be found, if not in the protocol. Alternatively, an explanation of why a DMC is not needed	______N/A______
	21b	Description of any interim analyses and stopping guidelines, including who will have access to these interim results and make the final decision to terminate the trial	______N/A______
Harms	22	Plans for collecting, assessing, reporting, and managing solicited and spontaneously reported adverse events and other unintended effects of trial interventions or trial conduct	______N/A______
Auditing	23	Frequency and procedures for auditing trial conduct, if any, and whether the process will be independent from investigators and the sponsor	______N/A______
**Ethics and dissemination**			
Research ethics approval	24	Plans for seeking research ethics committee/institutional review board (REC/IRB) approval	______18_______
Protocol amendments	25	Plans for communicating important protocol modifications (eg, changes to eligibility criteria, outcomes, analyses) to relevant parties (eg, investigators, REC/IRBs, trial participants, trial registries, journals, regulators)	______N/A______

For peer review only - http://bmjopen.bmj.com/site/about/guidelines.xhtml

Consent or assent	26a	Who will obtain informed consent or assent from potential trial participants or authorised surrogates, and how (see Item 32)	______18_______
	26b	Additional consent provisions for collection and use of participant data and biological specimens in ancillary studies, if applicable	______18_______
Confidentiality	27	How personal information about potential and enrolled participants will be collected, shared, and maintained in order to protect confidentiality before, during, and after the trial	______18_______
Declaration of interests	28	Financial and other competing interests for principal investigators for the overall trial and each study site	______21_______
Access to data	29	Statement of who will have access to the final trial dataset, and disclosure of contractual agreements that limit such access for investigators	______20_______
Ancillary and post-trial care	30	Provisions, if any, for ancillary and post-trial care, and for compensation to those who suffer harm from trial participation	______N/A______
Dissemination policy	31a	Plans for investigators and sponsor to communicate trial results to participants, healthcare professionals, the public, and other relevant groups (eg, via publication, reporting in results databases, or other data sharing arrangements), including any publication restrictions	______19_______
	31b	Authorship eligibility guidelines and any intended use of professional writers	_______N/A_____
	31c	Plans, if any, for granting public access to the full protocol, participant-level dataset, and statistical code	_______20______
**Appendices**			
Informed consent materials	32	Model consent form and other related documentation given to participants and authorised surrogates	_Suppl. file B____
Biological specimens	33	Plans for collection, laboratory evaluation, and storage of biological specimens for genetic or molecular analysis in the current trial and for future use in ancillary studies, if applicable	______10, 18____

*It is strongly recommended that this checklist be read in conjunction with the SPIRIT 2013 Explanation & Elaboration for important clarification on the items. Amendments to the protocol should be tracked and dated. The SPIRIT checklist is copyrighted by the SPIRIT Group under the Creative Commons "Attribution-NonCommercial-NoDerivs 3.0 Unported" license.

For peer review only - http://bmjopen.bmj.com/site/about/guidelines.xhtml

MRCZ/A/2899

LONDON SCHOOL of HYGIENE &TROPICAL MEDICINE

Investigating point-of-care diagnostics for sexually transmitted infections and antimicrobial resistance in primary care in Zimbabwe (IPSAZ)

STI TESTING

Principal Investigator: Dr Kevin Martin [MBBS, MRCP, MSc, DTM&H, DFSRH]
Phone number(s): +263 (0)774 410908

What you should know about this research study:

- We give you this consent form so that you may read about the purpose, risks, and benefits of this research study.
- Routine care is based upon the best known treatment and is provided with the main goal of helping the individual patient. The main goal of research studies is to gain knowledge that may help future patients.
- We cannot promise that this research will benefit you.
- You have the right to refuse to take part, or agree to take part now and change your mind later.
- Whatever you decide, it will not affect your regular care.
- Please review this consent form carefully. Ask any questions before you make a decision.
- Your participation is voluntary.

PURPOSE

We are conducting a research study to find out if it is feasible to offer testing for multiple sexually transmitted infections (STIs) and mental health screening to pregnant women when they attend clinic for routine care. We also want to know the proportion of pregnant women who have STIs and are at risk of depression.. You have been invited to take part because you are pregnant and attending clinic for antenatal care. We are hoping to recruit about 1,000 pregnant women in Harare into this study.

MEDICAL RESEARCH COUNCIL OF ZIMBABWE
10 DEC 2022
APPROVED
20 CAMBRIDGE ROAD, AVONDALE, HARARE

For peer review only - http://bmjopen.bmj.com/site/about/guidelines.xhtml

PROCEDURES AND DURATION

If you decide to participate, we will perform tests for the following STIs; chlamydia; gonorrhoea, trichomoniasis; syphilis; hepatitis B; and HIV. These are all infections that can be transmitted by sex. Syphilis and HIV testing is part of routine pregnancy care. Chlamydia, gonorrhoea, trichomoniasis, and syphilis, can all be cured with antibiotic treatment. If left untreated, these STIs can cause harm to both your baby and yourself. Hepatitis B is a virus that infects the liver, and can lead to liver damage. There is no cure, but specialist treatment may be available. Hepatitis B can be passed on to a baby at birth, but the risk of this can be reduced by giving a hepatitis B vaccine to the baby shortly after birth. HIV is a virus which weakens your immune system and your ability to fight everyday infections and disease. If left untreated, it can lead to AIDS, which is the name used to describe a number of potentially life-threatening infections and illnesses that happen when your immune system has been severely damaged by HIV. HIV can also be passed on to the baby during pregnancy. There's currently no cure for HIV, but there are very effective drug treatments that enable most of those on treatment to live a long and healthy life, to reduce the risk of passing the infection to your baby, and to prevent infection of sexual partners.

To perform the tests, we will require two vaginal swabs, which may be taken by yourself or by a member of the study team. We will also take a small fingerprick blood sample. The results for the trichomoniasis, syphilis, hepatitis B, and HIV tests, should all be ready within 30 minutes. During this time, you will undergo a questionnaire. We will ask questions about yourself, your pregnancy, STIs, your risk of STIs and screen for depression. You may stop at any point if you get tired or the questions make you uncomfortable.

The results for the chlamydia and gonorrhoea tests will take 90 minutes. We will ask you to wait or return later today to receive your chlamydia and gonorrhoea results. If you are unable to return to collect your results, and your results are positive, you will be contacted by telephone and asked to return.

If your results for chlamydia, gonorrhoea, trichomoniasis, or syphilis, are positive, we will offer you treatment free of charge. We will also advise that any sexual partners you have also receive treatment. This is important to both protect your partner, and also to prevent re-infection of you. We will provide a slip of paper you can give to your partner, and discuss any concerns you may have. We will <u>not</u> inform your partner of your results without your permission.

If you are newly diagnosed with HIV, we will follow routine practices at the clinic for referral to specialist services. If you screen positive for depression, will refer you to counselling services.

If you are newly diagnosed with hepatitis B, we will refer you to see a liver specialist. To help the specialist decide on the best management, we will also take a blood sample from the vein to check the levels of hepatitis B, and for liver damage. We will also try to arrange for a hepatitis B vaccine to be administered to your new-born baby, to prevent your baby from getting hepatitis B.

If your results for gonorrhoea are positive, a member of the study team will ask to take a further swab from your cervix, which is inside your vagina. These samples will be taken to the laboratory in Harare and be used to grow the gonorrhoea. This will be done right away. Tests will then be performed to check if the gonorrhoea is resistant to commonly used antibiotics. This is helpful for deciding what antibiotics should be recommended in guidelines.

For peer review only - http://bmjopen.bmj.com/site/about/guidelines.xhtml

For other tests to check resistance to antibiotics, the samples are stored in the laboratory in Zimbabwe during the study and tested at the end of the study. For some tests, including analysing the genes from the STIs, this may involve shipping your samples to a study laboratory outside Zimbabwe. If you are ok with this, an additional swab will be taken for these tests. Any of your samples sent to an external laboratory will be destroyed on completion of all the necessary tests. Destruction usually happens at the end of the study.

Importantly, you can participate in the study, but decline this part or other parts if you do not wish to undertake all of the procedures.

Finally, we will contact you by telephone after your baby is due to be born to find out information about your pregnancy, on partner notification, any thoughts on the testing processes, and to perform a repeat mental health screening. We will also check your health records and birth registers to gather additional information such as birth weight and delivery type.

RISKS AND DISCOMFORTS

Some of the topics that we discuss may be personal, and may bring up memories or feelings that you find upsetting or difficult. You can contribute as you wish, and there is no obligation to answer any question that you do not want to. Refusing to take part in some or all the questionnaires will not affect any services that you receive in any way, and we are not going to tell anyone the answers to your questions or what we have talked about.

BENEFITS AND/OR COMPENSATION

Testing may reveal that you have an STI. STIs can sometimes lead to problems in pregnancy, or infection of the new-born baby. Therefore, by testing for STIs we can offer treatment to reduce the chances of this happening. Treatment will be offered for free for chlamydia, gonorrhoea, trichomoniasis, and syphilis. If diagnosed with hepatitis B, we will refer you to a specialist for further assessment, and try to arrange for a hepatitis B vaccine to be administered to your new-born baby, to prevent your baby from getting hepatitis B.

This project will hopefully benefit others by providing important information on strategies to test for STIs in pregnancy. We cannot and do not guarantee or promise that you will receive any benefits from this study.

ALTERNATIVES TO PARTICIPATION

If you decide not to participate in this study, you will still receive routine pregnancy care. This includes testing for HIV and syphilis. Tests for chlamydia, gonorrhoea, trichomoniasis, and hepatitis B are not available in routine care in Zimbabwe.

Additionally, if you have symptoms of an STI and decide not to participate, you will still receive treatment to cover the possible causes of your symptoms in routine care.

For peer review only - http://bmjopen.bmj.com/site/about/guidelines.xhtml

CONFIDENTIALITY

If you agree to take part in this study by signing this document, all information obtained will be stored using a study number (instead of your name), in safe paper and computer files. No one will be able to access the information about you except for the research team.

We require your telephone number so that we can contact you after the birth to collect more information. Additionally, we may need to contact you if you have any positive results, but are unable to receive them on the same day. Contact details, and any other identifiable information, will be kept separately and will only be accessible to the research team.

With your permission, the healthcare team who provide your care at the clinic will be informed of your STI test results, so that your clinic records can be updated.

Any information that is obtained in connection with this study that can be identified with you will remain confidential and will be disclosed only with your permission. Under some circumstances, the MRCZ may need to review patient records for compliance audits.

ADDITIONAL COSTS

We hope to give you all of your results at the clinic on the same day as testing. If we are unable to do this, and you test positive for an STI, we will advise you to return to the clinic for treatment. Additionally, if we grow gonorrhoea in the lab that is resistant to the antibiotic we gave you, we will advise you to return to the clinic. We will cover the costs of treatment, but we will not be able to cover transport costs to reach the clinic.

DATA SHARING

Anonymised data from this study may be shared via a public data repository or by sharing directly with other researchers. You will not be identifiable by this information.

VOLUNTARY PARTICIPATION

Participation in this study is voluntary. If you decide not to participate in this study, your decision will not affect your future relations with any hospitals, clinics or other health services, nor with any health personnel or with the Biomedical Research and Training Institute or with the London School of Hygiene and Tropical Medicine. You are free to withdraw your consent and stop your involvement at any time without penalty.

For peer review only - http://bmjopen.bmj.com/site/about/guidelines.xhtml

FORM IPSAZ_ENGLISH_CONSENT: TESTING

Page 5 [of 5]

IRB No. MRCZ/A/2899

SIGNATURE PAGE

Investigating point-of-care diagnostics for sexually transmitted infections and antimicrobial resistance in primary care in Zimbabwe (IPSAZ)

STI TESTING

Protocol Version 1.2 dated 10 May 2022

OFFER TO ANSWER QUESTIONS

Before you sign this form, please ask any questions on any aspect of this study that is unclear to you. You may take as much time as necessary to think it over.

AUTHORIZATION

You are making a decision whether or not to participate in this study. Your signature indicates that you have read and understood the information provided above, have had all your questions answered, and have decided to take part.

- I have read the information concerning this study and I understand what will be required
- I understand that at any time I can withdraw from this study without giving a reason
- I understand that data about/from me may be shared via a public data repository or by sharing directly with other researchers, and that I will not be identifiable from this information.

- I agree to have samples from my vagina or cervix, and any gonorrhoea grown from my samples, stored, and shipped to a laboratory outside Zimbabwe for tests mentioned in this consent form (*Mark either "Yes" or "No" with your initials to indicate your choice*)

Yes ☐ **No** ☐

Name of Participant (print)	*Signature of Participant*	☐☐/☐/20☐☐ *Date*
Name of Staff obtaining consent	*Signature of Staff*	☐☐/☐/20☐☐ *Date*
Name of Witness (if required)	*Signature of Witness*	☐☐/☐/20☐☐ *Date*

YOU WILL BE OFFERED A COPY OF THIS CONSENT FORM TO KEEP.

If you have any questions concerning this study or consent form beyond those answered by the investigator, including questions about the research, your rights as a research participant or research-related injuries; or if you feel that you have been treated unfairly and would like to talk to someone other than a member of the research team, please feel free to contact the Medical Research Council of Zimbabwe (MRCZ) on telephone (04)791792 or (04) 791193 and cell phone lines 0784 956 128. The MRCZ Offices are located at 20 Cambridge Road, Avondale, Harare. This consent form has been reviewed and approved by the MRCZ, the Biomedical Research and Training Institute Institutional Review Board, and the London School of Hygiene & Tropical Medicine Interventions Research Ethics Committee.

MEDICAL RESEARCH COUNCIL OF ZIMBABWE
10 DEC 2022
APPROVED
20 CAMBRIDGE ROAD, AVONDALE, HARARE

For peer review only - http://bmjopen.bmj.com/site/about/guidelines.xhtml
